# CTLA-4 expression on CD4^+^ lymphocytes in patients with sepsis-associated immunosuppression and its relationship to mTOR mediated autophagic–lysosomal disorder

**DOI:** 10.3389/fimmu.2024.1396157

**Published:** 2024-07-22

**Authors:** Wei Cheng, Jiahui Zhang, Dongkai Li, Xianli Lei, Hao Wang, Na Cui

**Affiliations:** ^1^ Department of Critical Care Medicine, State Key Laboratory of Complex Severe and Rare Diseases, Peking Union Medical College Hospital, Chinese Academy of Medical Science and Peking Union Medical College, Beijing, China; ^2^ Department of Critical Care Medicine, Beijing Jishuitan Hospital, Capital Medical University, Beijing, China

**Keywords:** CTLA-4, CD4^+^ lymphocyte, mTOR, autophagic–lysosomal disorder, sepsis-associated immunosuppression

## Abstract

**Background:**

The aim of this study was to clarify the relationship between expression level of CTLA-4 on CD4^+^ T cells and sepsis-associated immunosuppression (SAI), and to elucidate the possible mechanism of mTOR pathway mediated autophagic-lysosomal disorder in regulating CTLA-4 expression.

**Methods:**

We enrolled 63 sepsis patients admitted to our ICU between January 1 and June 30, 2023. Peripheral blood mononuclear cells were isolated from the patients within 24 hours of recruitment. Expression levels of mTOR, P62, LC3II, and CTLA-4 on circulating CD4^+^ T lymphocytes were quantitated using flow cytometry. The association of these markers and relationship between CTLA-4 expression and the incidence of SAI and 28-day mortality were comprehensively analyzed.

**Results:**

Compared with non-immunosuppressed patients with sepsis, patients with SAI had a higher 28-day mortality rate (37.5% vs 13.0%, P=0.039) and higher CTLA-4 mean fluorescence intensity (MFI) on CD4^+^ T cells (328.7 versus 78.7, P<0.0001). CTLA-4 MFI on CD4^+^ cells was independently associated with the occurrence of SAI (95% confidence interval: 1.00–1.14, P=0.044). In patients with sepsis and SAI, non-survivors had higher CTLA-4 expression than survivors (sepsis: 427.5 versus 130.6, P=0.002; and SAI: 506.7 versus 225.2, P<0.0001). The sensitivity and specificity of CTLA-4 MFI at predicting 28-day mortality in patients with SAI was 100% and 80% respectively with the cutoff value of 328.7 and the area under the curve of 0.949. The MFI of mTOR, P62, and LC3II on CD4^+^ T cells were statistically higher in patients with SAI than in non-immunosuppressed patients (267.2 versus 115.9, P<0.0001; 314.8 versus 173.7, P<0.0001; and 184.7 versus 1123.5, P=0.012, respectively); P62 and LC3II were markedly higher in non-survivors than in survivors of sepsis (302.9 versus 208.9, P=0.039; and 244.3 versus 122.8, P<0.0001 respectively). The expression of CTLA-4 statistically correlated with that of LC3II in patients with sepsis, patients with SAI, and patients with SAI who did not survive (correlation coefficient: 0.69, 0.68, and 0.73, respectively, P<0.0001).

**Conclusions:**

CTLA-4 overexpression on CD4^+^ T cells was markedly associated with the incidence of SAI and had great relevance to 28-day mortality. mTOR pathway mediated autophagic-lysosomal disorder showed significant association with CTLA-4 expression.

## Introduction

Sepsis remains a significant global health challenge with mortality rate of over 10% and up to 40% when patients progressed into septic shock (1–3). An effective host response is critical, but subsequent life-threating cytokine release syndrome mediates less immediate benefit than collateral damage, and targeted immunomodulation or nonspecific immunosuppression can improve the prognosis of certain patients (4, 5). However, immunosuppression caused by a dysregulated host response is another key cause of increased mortality, and the role of immune augmentative therapy for these patients has been inconclusive (6–10). It is important to explore the underlying mechanisms of sepsis-associated immunosuppression (SAI) and identify possible intervention points.

Cytotoxic T lymphocyte antigen-4 (CTLA-4) is a co-inhibitory receptor on lymphocytes. CTLA-4 is expressed in regulatory and activated T cells and is mainly localized in the cytoplasm, acting on the cell surface. Endocytosis and lysosomal degradation are the process of movement, recycling and degradation (11). Enhanced CTLA-4 expression occurs more frequently in patients with sepsis (12), and blocking CTLA-4 improved survival in experimental bacterial and fungal sepsis (13, 14). In a phase I clinical study, anti-PD-1, a negative co-stimulatory molecule that acts in a fashion similar to CTLA-4 to suppress T cell function, was proved to be safe and effective based on immune biomarkers. However, subsequent immune modulatory therapies for sepsis did not yield satisfactory outcomes (15, 16). Inflammation is a highly sophisticated, complex response that is delicately controlled by the expression of positive and negative co-stimulatory molecules, so directly boosting the immune system may not be an appropriate treatment strategy.

Autophagy is a recognized lysosomal degradation pathway (17). Mammalian target of rapamycin (mTOR) is an atypical serine/threonine protein kinase that plays an important regulatory role in cell proliferation, metabolism, regeneration, and differentiation (18). Our team recently found that the mTOR pathway plays a critical role in the regulation of CD4^+^ T cell apoptosis during sepsis by improving autophagosome–lysosome fusion, and activation of the mTOR pathway may contribute to sepsis-induced myocardial dysfunction through the regulation of autophagy in cardiomyocytes (19, 20). It was found that the expression of CTLA-4 in meningioma tumor cells was consistently elevated in those harboring a mutation that disrupts the PI3K–AKT–mTOR pathway, and anti-CTLA-4 treatment inhibited autophagosome formation (21, 22). While cellular apoptosis and deficient autophagy are two of the most well-described mechanisms contributing to SAI (23) and based on the previous findings, we hypothesized that CTLA-4 expression on CD4^+^ T cells could be modulated by the mTOR pathway mediated autophagic–lysosomal disorders and play a key role in the development and prognosis of SAI.

This study was designed to illustrate the association between CTLA-4 expression on CD4^+^ lymphocytes and the occurrence of SAI and prognosis of patients with SAI, and furthermore to clarify the relationship between mTOR pathway mediated autophagic–lysosomal disorder and CTLA-4 expression.

## Materials and methods

### Study population

This prospective study was performed at Peking Union Medical College Hospital (PUMCH) from January 1, 2023 to June 30, 2023. Patients admitted to the intensive care unit (ICU) were enrolled when the following criteria were met: (1) aged over 18 years old; (2) ICU stay of more than 24 hours; and (3) met the definition of sepsis, in accordance with the 2016 American Society of Critical Care Medicine and European Society of Critical Care criteria. Sepsis was defined as life-threatening organ dysfunction caused by a dysregulated host response to infection, and organ dysfunction was identified as an acute change in total Sequential Organ Failure Assessment (SOFA) score of ≥2 points. Septic shock was defined as the clinical construct of sepsis with persisting hypotension requiring vasopressors to maintain a mean arterial pressure ≥65 mmHg and a serum lactate level >2 mmol/L despite adequate volume resuscitation (2, 6). Infection was defined on the basis of clinical features, laboratory findings, and imaging tests according to the criteria of the international sepsis forum consensus conference on definitions of infection (24). Patients were carefully excluded when they met any one of the following criteria: (1) aged less than 18 years old; (2) pregnant or breastfeeding; (3) receiving chemotherapy, radiation, or targeted therapy for unhealed tumors; (4) receiving glucocorticoids or immune suppressants for autoimmune disease; (5) acquired immunodeficiency, or blood system diseases involving white blood cells; or (6) patients who did not resuscitate or end-stage patients who were admitted for palliative care only. Non-septic patients in control group were those who undergone major surgery and admitted to our ICU during the study period. The study was approved by the PUMCH institutional review board (approval number: K3148- K22C2845). Consent forms were obtained from all the patients or the patients’ next of kin at ICU admission.

### Study method

Baseline characteristics (including age, sex, and comorbidities), vital signs and laboratory tests (including peripheral blood cell counts, biochemical test, arterial and venous blood gas analysis, and infectious parameters), and life-sustaining treatments (including mechanical ventilation, vasopressors, renal replacement therapy, and mechanical circulation support) on the first day of ICU admission were recorded. Acute Physiology and Chronic Health Evaluation (APACHE) II and SOFA scores were calculated. The patients were followed up 28 days after enrollment and were divided into survivors and non-survivors according to 28-day mortality. The lengths of ICU stay and in-hospital stay were also calculated.

Peripheral blood samples were collected in EDTA anticoagulant tubes on the first day following ICU admission. After immediate centrifugation, peripheral blood mononuclear cells (PBMCs) were isolated using the Ficoll density-gradient separation method. According to the manufacturer’s instructions, antibodies and their isotype controls were incubated with PBMCs at 4°C for 30 min, and the PBMCs were washed with staining buffer 3 times. Flow cytometric analysis was performed with a flow cytometer (BD Biosciences), and the results were analyzed using FlowJo software (V.10.1r5, TreeStar). Lymphocytes were gated based on side scatter and forward scatter, and the subsets of T cells (CD3^+^), and CD4^+^, Th1, Th2, Th17, Treg, and CD8^+^ T cells were quantitated. CD4^+^ lymphocytes were isolated and immunoassayed with combinations of fluorochrome-conjugated antibodies. Fluorescently labeled antibodies used for flow cytometry were purchased from BioLegend (San Diego, CA) and Invitrogen (California, United States) (antibodies used in the study: APC anti-human CD4, BioLegend 9808; LC3B Polyclonal Antibody, Invitrogen PA1-16930; SQSTM1 Polyclonal Antibody, Invitrogen PA5-27247; F(ab’)2-Donkey anti-Rabbit IgG (H+L) Secondary Antibody, PE, eBioscience™ Invitrogen 12-9718-42; Phospho-mTOR (Ser2448) Monoclonal Antibody (MRRBY), PE, eBioscience™ Invitrogen 12-9718-42; CD152 (CTLA-4) Monoclonal Antibody (14D3), PE, eBioscience™ Invitrogen 12-1529-42; Mouse IgG2a kappa Isotype Control (eBM2a), PE, eBioscience™ Invitrogen 12-4724-82). To measure intracellular cytokines in CD4^+^ T cells following ex vivo stimulation, cells were first stimulated in complete RPMI 1640 (Thermo Fisher Scientific) for 4 hours at 37°C with 500ng/ml PMA, 1ug/ml ionomycin, and 10ug/ml brefeldin A (all Sigma-Aldrich). For all intracellular cytokine staining, surface-stained cells were fixed and permeabilized (20min at 4°C) using Fixation/Permeabilization Solution before washes in Perm/Wash buffer (both BD Biosciencces). Cells were then stained with intracellular Antibodies as above except in Perm/Wash buffer. Appropriate isotype controls were included. For FACS analysis, events were acquired on a LSRFortessa (BD Biosciences) before analysis with FlowJo. The mean fluorescence intensities (MFI) of mTOR, P62, CTLA-4 and microtubule-associated protein light chain 3 type II (LC3II) were further quantitated on CD4^+^ T cells. 10,000 cells were analyzed from each sample.

SAI manifested various characteristics. As lymphocyte depletion was a main mechanism and correlated with poor prognosis, we divided the patients with sepsis in our study into two groups based on whether their circulating lymphocyte count exceeded 1,000/μL (10, 25–28). SAI was diagnosed when lymphocyte count was less than 1,000/uL at ICU admission. The expression of CTLA-4, mTOR, P62, and LC3II on CD4^+^ lymphocytes was comprehensively compared, as were length of ICU stay, length of hospital stay, and 28-day mortality rate, between the two groups. From June 1 to June 30, 2023, PBMC flow cytometric analysis and CD4^+^ lymphocyte immunostaining of the enrolled patients with sepsis was monitored on day 1 (ICU admission), day 3, and day 7. Non-septic critically ill patients during the same period were enrolled as the control group. The study was designed as a prospective clinical observational study without treatment strategy intervention. All of the regimens for patients were chosen by clinicians following the same principles as those recommended in the Surviving Sepsis campaign guidelines (1, 29).

### Statistics

Normally distributed data were presented as mean ± standard deviation, and significant differences were determined by Student’s t test. Non-normally distributed data were presented as median and interquartile range (IQR) and were compared using the Mann-Whitney U test. Categorial variables were expressed as proportions and analyzed using the chi-squared test or Fisher’s exact test. Multivariate logistic regression analyses were performed to determine the predictive value of CTLA-4 for the incidence of SAI. Receiver operating characteristic (ROC) curves were used to distinguish the diagnostic value of the tested parameters for SAI or 28-day mortality. Pearson’s analysis was performed to explore the association between the expression levels of CTLA-4 on CD4^+^ lymphocytes and biomarkers involved in the mTOR signaling pathway-autophagic lysosomal disorder axis. The significance level was set at a 2-sided P value of 0.05. Statistical analyses were performed using SPSS 25.0 (Chicago, Illinois).

## Results

### Baseline characteristics of enrolled patients with sepsis and comparison with non-septic critically ill patients

During the study period, 63 patients with sepsis were enrolled (**Additional File S1**), with a median age of 62 years (IQR: 51–71), of whom 57.1% were male (36 cases). Compared with non-septic critically ill patients, septic patients had significantly higher APACHE II and SOFA scores (19 versus 8, P<0.0001, and 8 versus 5, P=0.012, respectively), higher serum lactate levels (4.2 versus 2.0 mmol/L, P<0.0001) on admission, longer ICU stays (12 versus 4 days, P=0.002), and higher 28-day mortality (28.6% versus 0%, P=0.012). There were no statistical differences in lymphocyte counts and CD4^+^ T lymphocyte counts between the two groups (632 versus 738/μL, P=0.585, and 261 versus 287/μL, P=0.795, respectively), but the percentages of circulating CD4^+^ T lymphocytes expressing interleukin (IL)-6 and IL-17 were significantly higher in patients with sepsis (8.2% versus 5.1%, P=0.029; and 8.6% versus 7.0%, P=0.049, respectively). The MFI of CTLA-4, LC3II, mTOR, and P62 on CD4^+^ cells were significantly higher in patients with sepsis than in non-septic patients (185.5 versus 104.4, P<0.0001; 152.3 versus 99.6, P=0.001; 166.8 versus 143.6, P=0.029; and 244.0 versus 177.2, P=0.001, respectively) (**Additional File S2**).

### Comparison of patients with SAI and non-immunosuppressed septic patients

Patients with sepsis were divided into non-immunosuppressed and immunosuppressed (SAI) groups based on whether their circulating lymphocyte counts exceeded 1,000 cells/μL. Compared with the non-immunosuppressed patients, patients in the SAI group were older (median and IQR for age: 67 [58, 74] versus 57 [48, 69] years, P=0.021) and included a higher proportion with chronic kidney disease (17.5% versus 0%, P=0.033). Patients with SAI had longer ICU stays (13 versus 11 days, P=0.040) and a higher 28-day mortality rate (37.5% versus 13%, P=0.039). There were no statistical differences in sex, infection site, APACHE II score, SOFA score, or hospital stay (**Table 1**). Patients with SAI had higher serum lactate and creatinine levels at ICU admission (4.5 versus 3.0 mmol/L, P=0.046; 103.5 versus 73.0 μmol/L, P=0.013), and a higher proportion of immunosuppressed patients received continuous renal replacement therapy (37.5% versus 13.0%, P=0.039) and initial anti-fungal drug treatment (47.5% versus 8.7%, P=0.002) (**Additional File S3**).

**Table 1:** Comparison of baseline characteristics between patients with sepsis-associated immunosuppression and non-immunosuppressed septic patients.

	All (N=63)	Patients with SAI* N=40	Non-immuno-suppressed patients N=23	P
Age	62 (51, 71)	67 (58, 74)	57 (48, 69)	0.021
Sex	36 (57.1%)	21 (52.5%)	15 (65.2%)	0.326
Transferred from Ward Emergency Room	9 (14.3%) 54 (85.7%)	5 (12.5%) 35 (87.5%)	4 (17.4%) 19 (82.6%)	0.593
Comorbidities Chronic heart disease COPD Diabetes mellitus Chronic kidney disease Chronic liver disease Stable Solid Tumor	22 (34.9%) 5 (7.9%) 21 (33.3%) 7 (11.1%) 4 (6.3%) 17 (27.0%)	15 (37.5%) 5 (12.5%) 13 (32.5%) 7 (17.5%) 3 (7.5%) 12 (30.0%)	7 (30.4%) 0 (0%) 8 (34.8%) 0 (0.0%) 1 (4.3%) 5 (21.7%)	0.571 0.077 0.853 0.033 0.621 0.477
Infection sites Pulmonary Abdominal Urinary Tract Blood Stream Others	21 (33.3%) 33 (52.4%) 3 (4.8%) 3 (4.8%) 3 (4.8%)	15 (37.5%) 20 (50.0%) 2 (5.0%) 1 (2.5%) 2 (5.0%)	6 (26.1%) 13 (56.5%) 1 (4.3%) 2 (8.7%) 1 (4.3%)	0.756
Apache II score	19 (16, 28)	19 (16, 30)	19 (14, 25)	0.963
SOFA score	8 (5, 11)	8 (5, 11)	8 (6, 11)	0.884
ICU stay	12 (10, 16)	13 (11, 18)	11 (10, 13)	0.040
Hospital Stay	20 (16, 25)	21 (16, 26)	20 (16, 25)	0.639
28-day mortality	18 (28.6%)	15 (37.5%)	3 (13.0%)	0.039

*SAI Sepsis-associated immunosuppression, defined as sepsis patients with peripheral lymphocyte counts less than 1000/uL at ICU admission. Values are presented as median and interquartile range (IQR) for continuous variables or as number of cases and percentage for categorical data.

COPD, chronic obstructive pulmonary disease; Apache II score, acute physiology and chronic health evaluation II score; SOFA score, subsequent organ failure assessment score; ICU, intensive care unit.

**Figure 1:**
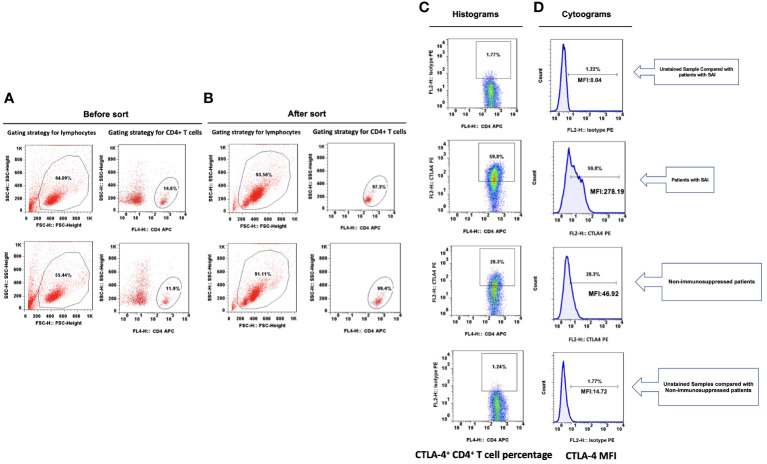
Representative flow dot plots of lymphocyte gating strategy The lymphocyte **(A)**, CD4^+^ T cell **(B)**, percentage of CTLA-4^+^ CD4^+^ T cells **(C)** and CTLA-4 MFI on CD4^+^ T cells **(D)** between patients with sepsis induced immunosuppression and non-immunosuppressed septic patients.

**Figure 2:**
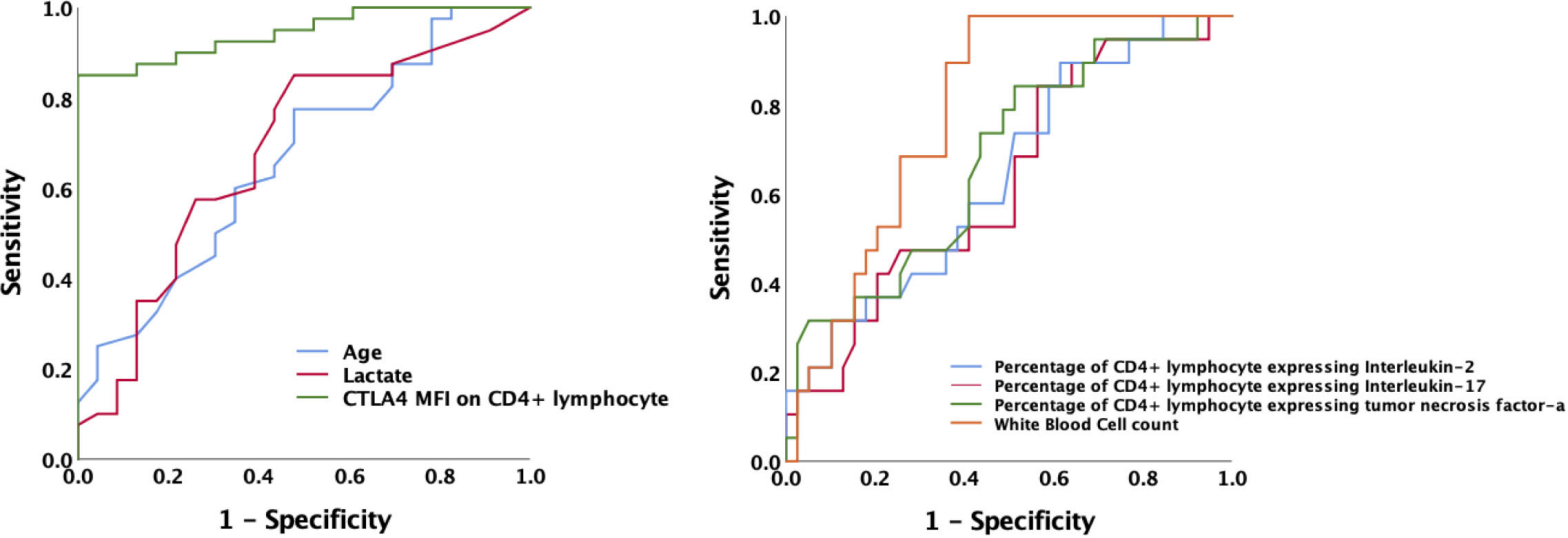
Receiver operating characteristic curve for predicting the occurrence of sepsis-associated immunosuppression in septic patients. AUCs: MFI of CTLA-4 on CD4^+^ T cells 0.945; age 0.662; lactate level 0.680; white blood cell counts 0.787; Percentage of CD4^+^ T lymphocyte expressing IL-2 0.644; Percentage of CD4^+^ lymphocyte expressing IL-17 0.630; Percentage of CD4^+^ lymphocyte expressing TNF-α 0.675.AUC, area under the curve; MFI, mean fluorescence intensity; CTLA-4, cytotoxic T lymphocyte antigen-4; IL, interleukin; TNF-α, tumor necrosis factor α.

Lymphocyte subsets were gated by side scatter and forward scatter, and then CD4^+^ lymphocytes were gated and immunoassayed with combinations of fluorochrome-conjugated antibodies. The percentages of CD4^+^ lymphocytes expressing mTOR, P62, CTLA-4, and LC3II, and the MFI of these markers on CD4^+^ lymphocytes, were further quantitated. 10,000 cells were analyzed from each sample (**Figure 1**). White blood cell, lymphocyte, T lymphocyte, CD4^+^ T lymphocyte, and CD8^+^ T lymphocyte counts were all lower in patients with SAI than in non-suppressed patients. The percentage of circulating CD4^+^ T lymphocytes expressing IL-2, IL-17, and tumor necrosis factor α (TNF-α) were significantly lower in patients with SAI (9.9% versus 13.6%, P=0.042; 7.9% versus 10.2%, P=0.044; and 9.4% versus 11.0%, P=0.042, respectively), whereas the percentage of CD4^+^ T lymphocytes expressing CTLA-4 was markedly higher in those with SAI (65.9% versus 45.8%, P<0.0001). When flow cytometry data were expressed as MFI, the MFI of CTLA-4, LC3II, mTOR, and P62 on CD4^+^ T lymphocytes were significantly higher in patients with SAI than in non-immunosuppressed patients (328.7 versus 78.7, P<0.0001; 184.7 versus 123.5, P=0.012; 267.2 versus 115.9, P<0.0001; 314.8 versus 173.7, P<0.0001 respectively) (**Table 2**). Multivariate logistic regression analysis was performed using factors that were significantly different in univariate analysis between immunosuppressed and non-immunosuppressed septic patients; CTLA-4 MFI on circulating CD4^+^ T lymphocytes proved to be an independent risk factor for the incidence of SAI (odds ratio 1.068, 95% confidence interval 1.00–1.14, P=0.044) (**Table 3**). To further determine the discriminatory ability of CTLA-4 for the incidence of immunosuppression in patients with sepsis, we generated ROC curves, and found that CTLA-4 MFI was better than the other parameters, with an area under the curve (AUC) value of 0.945 (**Figure 2**).

**Table 2:** Comparison of T lymphocyte subsets and markers on CD4^+^ T cells between patients with SAI and non-immunosuppressed sepsis patients.

	All sepsis patients N=63	Patients with SAI* N=40	Non-immunosuppressed sepsis patients N=23	P
T lymphocyte subsets (median and IQR) (/uL)				
White blood cell	11880 (7785, 16930)	9260 (6620, 14900)	15380 (11880, 19550)	0.003
Lymphocyte	6322 (394, 1158)	445 (360, 613)	1334 (1152, 1500)	<0.0001
B lymphocyte	119 (63, 216)	74 (44, 118)	249 (179, 341)	<0.0001
NK T cells	57 (32, 92)	42 (19, 60)	86 (60, 113)	0.01
T lymphocyte	433 (261, 884)	305 (224, 416)	940 (837, 1110)	<0.0001
CD4^+^ T cell	261 (152, 595)	178 (134, 257)	637 (537, 699)	<0.0001
CD8^+^ T cell	145 (92, 246)	108 (74, 145)	305 (196, 404)	<0.0001
CD4^+^CD28^+^ T cell	249 (139, 555)	166 (104, 233)	587 (550, 667)	<0.0001
CD8^+^CD28^+^ T cell	75 (33, 158)	38 (23, 76)	165 (132, 231)	<0.0001
CD8^+^CD38^+^ T cell	64 (45, 130)	56 (33, 84)	130 (74, 192)	<0.0001
CD4^+^CD25^+^ Treg	38 (23, 77)	30 (16, 40)	86 (62, 103)	<0.0001
Foxp3^+^ Treg	17 (10, 30)	13 (7, 17)	34 (21, 41)	<0.0001
CD4^+^/CD8^+^ T cell ratio	1.8 (1.2, 2.9)	1.7 (1.1, 2.9)	1.9 (1.3, 2.9)	0.253
Markers on CD4^+^ T cells (median and IQR)				
IFN-r (%^¥^)	7.4 (4.7, 9.1)	6.8 (4.4, 8.7)	8.4 (6.4, 10.2)	0.253
IL-2 (%)	11.7 (8.8, 16.3)	9.9 (8.1, 15.6)	13.6 (9.3, 18.2)	0.042
IL-6 (%)	8.2 (5.5, 11.2)	7.9 (5.8, 10.8)	8.6 (5.2, 12.5)	0.924
IL-17 (%)	8.6 (6.4, 12.0)	7.9 (5.9, 10.4)	10.2 (7.2, 12.6)	0.044
TNF-α (%)	10.5 (7.3, 12.6)	9.4 (6.9, 12.2)	11.0 (9.1, 15.1)	0.042
CTLA-4 (%)	56.7 (41.9, 77.6)	65.9 (49.7, 85.9)	45.8 (30.2, 51.8)	<0.0001
LC3II (%)	46.5 (25.3, 85.2)	55.9 (29.6, 88.8)	43.7 (21.6, 71.1)	0.341
mTOR (%)	81.5 (60.5, 91.3)	81.9 (65.9, 92.7)	81.5 (58.8, 90.0)	0.924
P62 (%)	95.7 (90.3, 97.6)	96.2 (93.5, 97.8)	94.8 (82.1, 96.6)	0.140
CTLA-4 (MFI)	185.5 (93.8, 366.4)	328.7 (188.6, 440.8)	78.7 (49.2, 129.5)	<0.0001
LC3II-MFI	152.3 (100.2, 236.7)	184.7 (105.7, 266.3)	123.5 (99.1, 153.2)	0.012
mTOR-MFI	166.8 (114.5, 303.3)	267.2 (139.8, 360.8)	115.9 (83.4, 159.7)	<0.0001
P62-MFI	244.0 (184.3, 340.0)	314.8 (250.8, 367.6)	173.7 (160.8, 194.9)	<0.0001

Values are presented as median and interquartile range (IQR) for continuous variables or as number of cases and percentage for categorical data. *SAI, Sepsis-associated immunosuppression, defined as sepsis patients with peripheral lymphocyte counts less than 1000/uL. ¥ means proportions of marker positive CD4^+^ lymphocytes.

IQR, interquartile range; NK T cells, natural killer T cells; IFN-r, interferon r; IL, interleukin; TNF-α, tumor necrosis factor α; CTLA-4, cytotoxic T lymphocyte antigen-4; LC3II, microtubule-associated protein light chain 3 type II; mTOR, mammalian target of rapamycin; MFI, mean fluorescence intensity.

**Table 3:** Multivariate logistic regression analysis of factors significantly different between patients with sepsis-associated immunosuppression and non-immunosuppressed septic patients.

	B	OR	95% CI	P
Age	-0.090	0.914	0.671, 1.25	0.571
Chronic Kidney Disease	-22.7	<0.001	<0.001, -	0.998
Lactate	-0.794	0.452	0.146, 1.402	0.169
CTLA-4 MFI	0.066	1.068	1.00, 1.14	0.044
White Blood Cell count	<0.001	1.000	0.999, 1.00	0.096
IL-17 (%^¥^)	-0.120	0.887	0.317, 2.49	0.820
IL-2 (%)	-0.519	0.595	0.295, 1.20	0.147
TNF-α (%)	0.142	1.153	0.502, 2.65	0.738

^¥^ means proportions of marker positive CD4^+^ lymphocytes.

OR, odds ratio; 95%CI, 95% confidential intervals; CTLA-4 MFI, the Mean Fluorescence Intensity of cytotoxic T lymphocyte antigen-4 on CD4^+^ lymphocyte; IL, interleukin; TNF-α, tumor necrosis factor α.

### Comparison between survivors and non-survivors among patients with SAI and patients with sepsis

**Figure 3:**
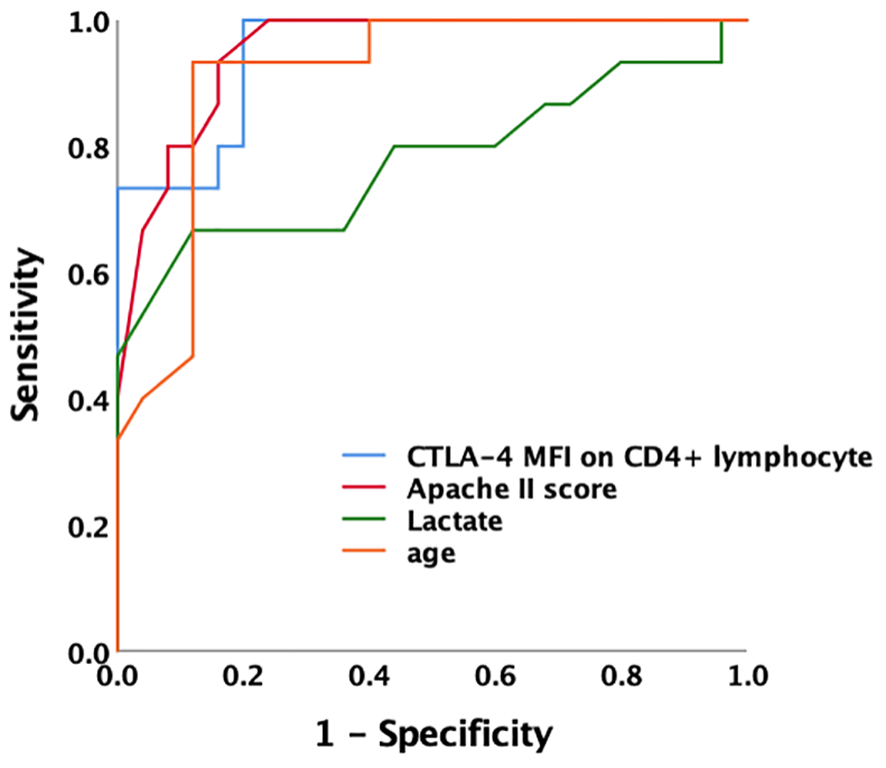
Receiver operating characteristic curve for predicting 28-day mortality of sepsis-associated immunosuppression the AUC of CTLA-4 MFI for prediction of 28-day mortality of patients with sepsis-associated immunosuppression was 0.949. A cutoff value of CTLA-4 MFI 328.7 at ICU admission was able to predict prognosis with a sensitivity of 100% and specificity of 80%. AUC of Apache II, lactate level and age was 0.952, 0.777 and 0.911 respectively. AUC, area under the curve; MFI, mean fluorescence intensity; CTLA-4, cytotoxic T lymphocyte antigen-4; ICU, intensive care unit; Apache II, acute physiology and chronic health evaluation II score.

We comprehensively compared survivors and non-survivors in the SAI group based on 28-day mortality. Non-surviving patients were older (73 versus 59 years old, P<0.0001) and had higher scores for critical illness (APACHE II score 30 versus 16, P<0.0001; SOFA score 11 versus 6, P=0.001). In the non-survivor group serum lactate levels were also higher (6.6 versus 3.9 mmol/L, P<0.0001). Among the non-surviving patients with SAI, the percentages of CD4^+^ T lymphocytes expressing IL-17, CTLA-4, and LC3II were markedly higher than among the survivors (9.3% versus 6.5%, P=0.009; 90.8% versus 56.7%, P<0.0001; and 88.8% versus 37.7%, P=0.009, respectively), whereas the percentage expressing TNF-α was lower (7.1% versus 10.5%, P=0.044). The MFI of CTLA-4 and LC3II on CD4^+^ lymphocytes were higher among the non-surviving patients with SAI than among the surviving patients (506.7 versus 225.2, P<0.0001; and 265.1 versus 145.9, P=0.01, respectively) (**Table 4**; **Additional Files S4, S5**). The ROC curve demonstrated that the AUC of CTLA-4 MFI for the prediction of 28-day mortality of patients with SAI was 0.949, which was comparable to APACHE II score (AUC: 0.952) and better than serum lactate level at admission (AUC: 0.777). A cutoff value of 328.7 for CTLA-4 MFI at ICU admission was able to predict 28-day mortality of patients with SAI with a sensitivity of 100% and a specificity of 80% (**Figure 3**).

**Table 4:** Factors significantly different between survivors and non-survivors in patients with sepsis-associated immunosuppression and in sepsis patients.

Comparison between survivors and non-survivors in patients with Sepsis-associated immunosuppression				
	All(N=40)	Non-survivors (N=15)	Survivors (N=25)	P
Baseline characters				
Age (years)	67 (58, 7,4)	73 (70, 77)	59 (50, 67)	<0.0001
Apache II score	19 (16, 30)	30 (27, 35)	16 (15, 19)	<0.0001
SOFA score	8 (5, 11)	11 (10, 12)	6 (4, 8)	0.001
Hospital Stay	21 (16, 26)	17 (14, 23)	22 (19, 27)	0.050
Lactate (mmol/L)	4.5 (3.5, 6.3)	6.6 (4.5, 9.9)	3.9 (3.3, 5.3)	<0.0001
Immunostaining markers on CD4^+^ T cells				
IL-17 (%^¥^)	7.9 (5.9, 10.4)	9.3 (8.4, 12.3)	6.5 (5.6, 9.5)	0.009
TNF-α (%)	9.4 (6.9, 12.2)	7.1 (6.7, 11.1)	10.5 (8.2, 13.4)	0.044
CTLA-4 (%)	65.9 (49.7, 85.9)	90.8 (74.4, 93.4)	56.7 (39.8, 64.4)	<0.0001
LC3II (%)	55.9 (29.6, 88.8)	88.8 (59.8, 91.9)	37.7 (18.2, 70.9)	0.009
CTLA-4 MFI	328.7 (188.6, 440.8)	506.7 (366.4, 571.5)	225.2 (152.0, 307.9)	<0.0001
LC3II-MFI	184.7 (105.7, 266.3)	265.1 (199.0, 312.5)	145.9 (78.3, 220.2)	0.01

Comparison between survivors and non-survivors in sepsis patients				
	All (N=63)	Non-survivors (n=18)	Survivors (n=45)	P
Baseline characters				
Immunosuppressed (%)	40 (63.5%)	15 (83.3%)	25 (55.6%)	0.039
Age (years)	62 (51, 71)	71 (70, 77)	58 (48, 67)	<0.0001
Apache II score	19 (16, 28)	30 (25, 35)	17 (14, 22)	<0.0001
SOFA score	8 (5, 11)	11 (10, 12)	7 (5, 11)	0.002
Hospital Stay	20 (16, 25)	18 (14, 21)	21 (18, 26)	0.015
Lactate (mmol/L)	4.2 (2.7, 6.0)	6.9 (4.5, 9.9)	3.6 (2.5, 4.8)	0.001
Procalcitonin (ng/ml)	4.0 (0.9, 19.0)	16.5 (4.2, 27)	2.7 (0.7, 13.5)	0.010
hsCRP (mg/L)	158.1 (81.4, 248.4)	228.5 (170.1, 320.2)	130.4 (75.9, 223.2)	0.002
T lymphocyte subset counts (/uL) (median and IQR)				
Lymphocyte	632 (394, 1158)	425 (375, 643)	797 (465, 1312)	0.015
T lymphocyte	433 (261, 884)	315 (227, 423)	597 (303, 900)	0.028
CD4^+^ T cell	261 (152, 595)	180 (145, 269)	401 (172, 612)	0.031
CD4^+^CD28^+^ T cell	249 (139, 555)	167 (1221, 249)	357 (164, 569)	0.015
CD8^+^CD28^+^ T cell	75 (33, 158)	36 (26, 60)	94 (42, 169)	0.003
Markers on CD4^+^ T cells (median and IQR)				
CTLA-4 (MFI)	185.5 (93.8, 366.4)	427.5 (346.9, 555.8)	130.6 (65.8, 230.1)	0.002
CTLA-4 (%)	56.7 (41.9, 77.6)	90.2 (73.2, 93.5)	50.3 (32.7, 59.1)	<0.0001
LC3II (%)	46.5 (25.3, 85.2)	87.7 (64.8, 91.3)	37.8 (19.4, 63.0)	<0.0001
LC3II-MFI	152.3 (100.2, 236.7)	244.3 (181.1, 307.3)	122.8 (90.1, 166.9)	<0.0001
P62-MFI	244.0 (184.3, 340.0)	302.9 (225.0, 381.7)	208.9 (174.3, 319.7)	0.039

Values are presented as median and interquartile range (IQR) for continuous variables or as number of cases and percentage for categorical data. ¥ means proportions of positive CD4^+^ lymphocytes.

Apache II score, acute physiology and chronic health evaluation II score; SOFA score, subsequential organ failure assessment score; hsCRP, hypersensitive C-reactive protein; IL-17, interleukin 17; TNF-α, tumor necrosis factor α; CTLA-4, cytotoxic T lymphocyte antigen 4; LC3II, microtubule-associated protein light chain 3 type II; MFI, mean fluorescence intensity.

Subsequently, all of the patients with sepsis were divided into surviving and non-surviving groups according to 28-day mortality. Like non-survivors among patients with SAI, non-survivors among patients with sepsis were older and more critically ill with higher lactate levels, with a higher percentage of CD4^+^ T lymphocytes expressing CTLA-4 and LC3II, and higher CTLA-4 and LC3II MFI on CD4^+^ lymphocytes than survivors of sepsis. In addition, non-surviving sepsis patients had higher procalcitonin and high-sensitivity C reactive protein levels (16.5 versus 2.7ng/mL, P=0.010; and 228.5 versus 130.4mg/L, P=0.002, respectively). The peripheral lymphocyte, T lymphocyte, CD4^+^ T cell, CD4^+^CD28^+^ T cell, and CD8^+^CD28^+^ T cell counts were also lower among the non-survivors with sepsis than among the survivors (425 versus 797, P=0.015; 315 versus 597, P=0.028; 180 versus 401, P=0.031; 167 versus 357, P=0.015; and 36 versus 94, P=0.003, respectively) (**Table 4**; **Additional File S6**). The AUC of CTLA-4 MFI for the prediction of 28-day mortality of all sepsis patients was 0.906, which was better than those for lymphocyte and CD4^+^ T lymphocyte counts (AUC: 0.650 and 0.693. respectively) (**Additional File S7**).

### Correlation between CTLA-4 and markers involved in mTOR pathway mediated autophagic–lysosomal disorder

**Figure 4:**
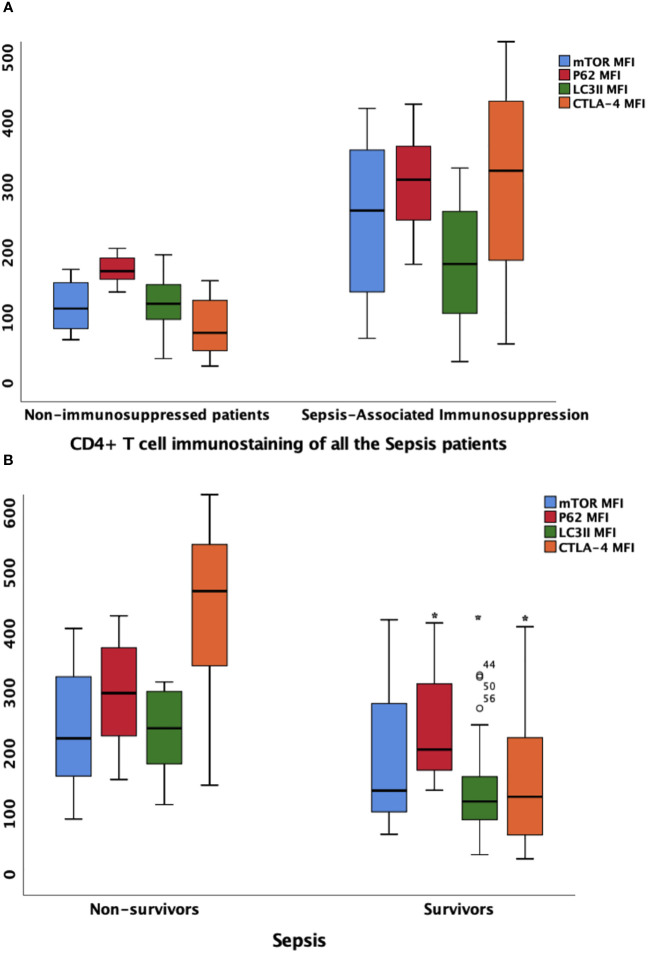
the MFIs of CTLA-4, mTOR, P62 and LC3II on CD4+ lymphocyte in different patient group **(A)** Comparison of marker MFIs on CD4^+^ lymphocyte between patients with sepsis-associated immunosuppression and non-immunosuppressed patients. The MFIs of mTOR, P62, LC3II and CTLA-4 were all significantly increased in patients with sepsis-associated immunosuppression (267.2 vs 115.9, P<0.0001; 314.8 vs 173.7 P<0.0001; 184.7 vs 123.5, P=0.012 and 328.7 vs 78.7, P<0.0001 respectively). **(B)** Comparison of marker MFIs on CD4^+^ lymphocyte between survivors and non-survivors of sepsis patients. Survivors and non-survivors were stratified according to 28-day mortality. Black Asteroids meant the comparison between the two groups was significant with P<0.05. The MFI s of P62, LC3II and CTLA-4 were significantly different between survivors and non-survivors of septic patients (302.9 vs 208.9, P=0.039, 244.3 vs 122.8, P<0.0001 and 427.5 vs 130.6, P=0.002 respectively). MFI, mean fluorescence intensity; mTOR, Mammalian target of rapamycin; LC3II, microtubule-associated protein light chain 3 type II; CTLA-4, cytotoxic T lymphocyte antigen-4.

The MFI of mTOR, P62, and LC3II on CD4^+^ T lymphocyte were higher in patients with SAI than in non-immunosuppressed patients (267.2 versus 115.9, P<0.0001; 314.8 versus 173.7, P<0.0001; and 184.7 versus 123.5, P=0.012, respectively). The percentage of CD4^+^ lymphocytes expressing CTLA-4 and CTLA-4 MFI on CD4^+^ cells were also significantly higher (65.9% versus 45.8%, P<0.0001; and 328.7 versus 78.7, P<0.0001, respectively) (**Table 2**, **Figure 4A**; **Additional File S8**).

Furthermore, we compared the expression intensity of biomarkers on CD4^+^ T lymphocytes between survivors and non-survivors based on 28-day mortality of all the patients with sepsis. Compared with those in survivors, the MFI of P62 and LC3II were higher in non-survivors (302.9 versus 208.9, P=0.039; and 244.3 versus 122.8, P<0.0001, respectively). In addition, the percentage of CD4^+^ cells expressing CTLA-4 and CTLA-4 MFI on CD4^+^ cells were also higher among non-surviving patients with sepsis (90.2% versus 50.3%, P<0.0001; and 427.5 versus 130.6, P=0.002, respectively) (**Figure 4B**; **Additional Files S6, S8**).

**Figure 5:**
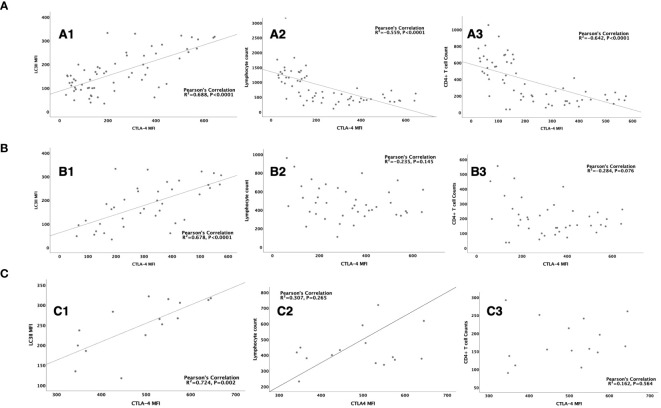
The correlation between CTLA-4 MFI on CD4+ lymphocyte and LC3II MFI, lymphocyte count and CD4+ T cell count **(A)**: The correlation between CTLA-4 MFI on CD4^+^ lymphocyte and LC3II MFI on CD4^+^ cells (R2 = 0.688, P<0.0001) (A1), lymphocyte count (R2= -0.559, P<0.0001) (A2), CD4^+^ T cell count (R2=-0.642, P<0.0001) (A3) respectively in sepsis patients. **(B)**: The correlation between CTLA-4 MFI on CD4^+^ lymphocyte and LC3II MFI on CD4^+^ cells (R2 = 0.678, P<0.0001) (B1), lymphocyte count (R2= -0.235, P=0.145) (B2), CD4^+^ T cell count (R2=-0.284, P=0.076) (B3) respectively in patients with sepsis induced immunosuppression. **(C)**: The correlation between CTLA-4 MFI on CD4^+^ lymphocyte and LC3II MFI on CD4^+^ cells (R2 = 0.678, P<0.0001) (C1), lymphocyte count (R2= -0.235, P=0.145) (C2), CD4^+^ T cell count (R2=-0.284, P=0.076) (C3) respectively in non-survived patients with sepsis induced immunosuppression. CTLA-4, cytotoxic T lymphocyte antigen-4; LC3II, microtubule-associated protein light chain 3 type II; MFI, mean fluorescent intensity.

Multivariate logistic regression showed that CTLA-4 MFI on CD4^+^ lymphocytes was an independent risk factor for the occurrence of SAI. When compared within the sepsis group or SAI group, CTLA-4 MFI on CD4^+^ lymphocytes from non-survivors was also significantly higher than that from survivors (**Table 4**; **Additional File S6**). As LC3II was also found to be significantly different, which was a biomarker of mTOR pathway mediated autophagic–lysosomal disorders, we hypothesized that this disorder might be associated with differing expression levels of CTLA-4 on CD4^+^ lymphocytes. Consistent with this, Pearson’s analysis identified that CTLA-4 overexpression significantly correlated with LC3II expression in patients with sepsis (R^2^ = 0.688, P<0.0001), patients with SAI (R^2^ = 0.678, P<0.0001), and non-surviving patients with SAI (R^2^ = 0.724, P=0.002) (**Figure 5**).

### Dynamic changes in CTLA-4 and correlation with incidence of SAI and prognosis of patients with SAI

**Figure 6:**
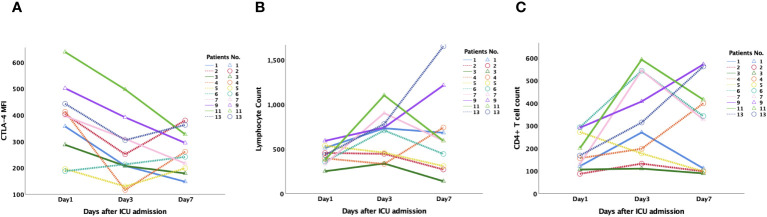
Dynamic changes of CTLA-4 MFI on CD4^+^ lymphocyte, peripheral lymphocyte count and CD4^+^ T cell count in patients with sepsis-associated immunosuppression. 13 patients were monitored dynamically, and 10 patients were diagnosed as sepsis-associated immunosuppression according to our grouping criteria. **(A)**: CTLA-4 MFI on CD4^+^ lymphocyte of survived patients (Pts No.1,3,7,9,11) steadily decreased from day 1 to day 7, while that of non-survived patients (Pts NO. 2,3,4,5,13) increased on day 7 after decreased on day 3. There was no significant tendency for the changes of lymphocyte **(B)** and CD4^+^ lymphocyte counts **(C)**. CTLA-4, cytotoxic T lymphocyte antigen-4; MFI, mean fluorescent intensity.

To further illustrate the connection between CTLA-4 and the incidence and 28-day mortality of SAI, PBMCs were collected from septic patients on days 1, 3, and 7 after enrollment following admittance to the ICU between June 1 and June 30, 2023. Thirteen patients were enrolled, and ten patients were diagnosed with SAI according to our grouping criteria. Patients with SAI had lower lymphocyte and CD4^+^ lymphocyte counts (**Additional File S9**). When grouped based on 28-day mortality, surviving patients with SAI had decreasing CTLA-4 MFI on CD4^+^ lymphocytes from day 1 to day 7, whereas non-surviving patients had increasing CTLA-4 MFI on day 7 after an initial decrease on day 3. There were no statistically significant tendencies among the changes in lymphocyte and CD4^+^ lymphocyte counts (**Figure 6**).

## Discussion

In our study, SAI was associated with a higher 28-day morality rate for patients with sepsis, and CTLA-4 MFI on CD4^+^ T lymphocytes was an independent risk factor for the incidence of SAI. CTLA-4 expression intensity on CD4^+^ lymphocytes was also a good predictor of 28-day mortality in patients with SAI, proving to be as effective as APACHE II score and lactate level. More importantly, a strong correlation was found between CTLA-4 expression on CD4^+^ lymphocytes and markers of the mTOR pathway mediated autophagic–lysosomal disorder, as expression of mTOR, P62, and LC3II was significantly different between patients with SAI and non-immunosuppressed patients; further, P62 and LC3II expression significantly differed between 28-day survivors and non-survivors. The expression of CTLA-4 significantly correlated with that of LC3II in patients with sepsis, patients with SAI, and non-surviving patients with SAI.

Sepsis was defined as organ dysfunction caused by a dysregulated host response, and this kind of dysregulation often manifests as immune suppression, which delays pathogen removal and subsequent infection control (2, 28). However, due to the heterogeneity of sepsis, there are currently no perfect indicators of this impaired immune function. Peripheral lymphocyte count has been commonly used to identify immunosuppression and predict the mortality of sepsis patients (25, 28, 30); however, lymphocyte counts can be affected by many factors, including body temperature (31) and correlated poorly with prognosis. In our study, CTLA-4 expression on CD4^+^ lymphocytes was a better predictor than peripheral lymphocyte count for the 28-day mortality of patients with SAI, with prognostic performance comparable to that of APACHE II score and higher than that of serum lactate level.

T cell activation is delicately controlled by the expression of positive and negative co-stimulatory molecules. CTLA-4 is one of the negative co-stimulatory molecules to suppress T cell function (32). In our study, overexpression of CTLA-4 on CD4^+^ lymphocytes was significantly associated with poor prognosis, consistent with the findings of Guignant et al., who showed PD-1 overexpression occurred on the circulating T cells of septic shock patients and correlated with increased mortality and immune dysfunction (33). Inhibiting the activity of negative co-stimulatory molecules helps to improve the T cell function and thus increase the immunity of septic patients, which could be a good approach for restoring dysregulated host response.

In mouse models of fungal sepsis, CTLA-4 blockade was found effective at improving survival (13). A phase I randomized controlled study proved the safety and pharmacokinetics of anti-PD-1 inhibitor in patients with SAI (15). Although anti-CTLA-4 antibody was already available, few reports or studies illustrated its effect on sepsis and SAI. Actually, after decades of research, no specific treatments to augment immune response have been validated for sepsis in clinical trials, and this hypothesis remains in the realm of speculation. Directly boosting instead of regulating the immune system in sepsis might not be appropriate (16, 34). Direct inhibition of co-inhibitory molecules could weaken the body’s ability to clear pathogens, which could instead cause further bodily deterioration. The current predicament for SAI treatment requires us to have a deeper understanding of the regulation mechanism of CTLA-4 and find more appropriate intervention sites, so as to achieve the most appropriate immune regulation of patients with sepsis and patients with SAI. The association between mTOR mediated autophagic lysosomal disorder and CTLA-4 expression on CD4+ T lymphocyte was of great promise.

The mTOR pathway is an evolutionarily conserved mechanism which primarily controls cell metabolism and survival. It was recently found that the mTOR pathway played vital role in regulation of CD4^+^ T-cell apoptosis through autophagic lysosomal fusion dysfunction during sepsis (19). Our study confirmed that CTLA-4 expression on CD4^+^ cells might also be modulated by mTOR pathway mediated autophagic lysosomal disorder. We demonstrated here that typical SAI was associated with increased CTLA-4 expression on CD4^+^ lymphocytes, and a marked correlation was found between CTLA-4 expression and a marker of mTOR pathway mediated autophagic–lysosomal disorder, LC3II; the accumulation of LC3II could be attributable to increased autophagosome formation or decreased lysosomal fusion and degradation. The correlation between CTLA-4 and LC3II indicated that the overexpression of CTLA-4 could be caused by autophagic lysosomal disorder. Putting the results of our study together, mTOR could exacerbate the poor outcome of SAI by inhibiting autophagosome-lysosome fusion, leading to impaired autophagy, and further causing CTLA-4 accumulation and exacerbating CD4+ T cell dysfunction. Thus, controlling the intensity of CTLA-4 on CD4^+^ T cells through modulating the upstream signal of autophagic–lysosomal fusion could be much more physiological. Just as illustrating the role of mTOR for regulating CD4^+^ lymphocyte apoptosis by modulating the endoplasmic reticulum stress (35), further animal and clinical trials are needed to validate this mechanism of mTOR mediated autophagic lysosomal disorder for CTLA-4 expression on CD4^+^ T cells.

Many advantages of this study are worth highlighting. First, the study not only confirmed the strong correlation between CTLA-4 and LC3II expression on CD4^+^ lymphocytes, but also tested the prognostic value of CTLA-4 for patients with SAI. Besides, via monitoring of dynamic changes in lymphocyte count, CD4^+^ T cell count and CTLA-4 MFI on CD4^+^ cells on days 1, 3, and 7 after enrollment, the correlation between CTLA-4 expression and prognosis was reconfirmed. We found the CTLA-4 MFI on the CD4^+^ lymphocytes of surviving patients steadily decreased from day 1 to day 7, but there was no significant tendency among the changes in lymphocyte and CD4^+^ lymphocyte counts. Second, many pro-inflammatory and immunosuppressive cytokines are produced during the response to infection, and serum levels of cytokines have been measured in various studies (13, 36, 37). However, circulating cytokine levels can be difficult to measure because of their short half-lives, and circulating levels may not accurately reflect local tissue levels. Previous studies (33, 38) showed no correlations between PD-1 expression on CD4^+^ lymphocytes and changes in serum IL-10 or PD-L1 concentrations. To avoid these influences, we instead tested the percentages of CD4^+^ lymphocytes producing cytokines and the MFI of each cytokine in CD4^+^ cells in our study. Finally, non-septic critically ill patients were chosen as a control group to further rule out the influences of non-infectious inflammation, which strengthens the results of this study.

Some limitations still need to be considered. First, although the correlation between CTLA-4 expression on CD4^+^ lymphocytes and immunosuppression, and the association between CTLA-4 expression and autophagic–lysosomal disorders, were identified among a large sample of patients with sepsis, this study was purely a clinical study. The causal relationship between CTLA-4 overexpression and autophagic disorders requires further basic studies or Mendelian randomization for confirmation. Second, we focused on the expression of co-inhibitory molecules and cytokines on CD4^+^ lymphocytes in this study, as CD4^+^ lymphocytes play vital roles in defense against bacterial infections. Recent studies found that PD-L1 expression on NK cells was associated with the prognosis of septic patients, and an elevated PD-1/CD28 ratio in CD8^+^ T cells predicted nosocomial infection in patients with sepsis (39, 40). The expression of CTLA-4 and its correlation with autophagic–lysosomal disorder should be further studied on CD8^+^ lymphocytes and NK cells. However, the role of CD8^+^ lymphocytes in bacterial sepsis is considered less central to the response, and the correlation between the expression of co-inhibitory molecules on CD8^+^ lymphocytes and prognosis has varied in different studies (33, 41). Third, sepsis is a complex disease with varying immune manifestations among individuals and at different points in the disease course. Therefore, we performed dynamic monitoring of CTLA-4 expression on CD4^+^ cells in our study to identify the relationship with prognosis. However, most patients in the study were examined only on the first day after ICU admission, and more frequent examination and dynamic monitoring of immune function during immunomodulatory therapy will be helpful to understand the role of CTLA-4 expression and the underlying mechanism in real time. At last, due to the high incidence of sepsis, relatively small sample was still a major limitation of the study, and the same trend of age with lymphocyte and CTLA-4 between the SAI and non-immunosuppressed group, the survived and non-survived group could further lower the credibility of the results. In the future, larger patient sample, clinical intervention research and animal experiment could test the association between CTLA-4 and mTOR pathway mediated autophagic-lysosomal disorder.

## Conclusions

CTLA-4 overexpression on CD4^+^ lymphocytes was markedly associated with the incidence of SAI and affected the prognosis of these patients. The expression of CTLA-4 on CD4^+^ lymphocytes significantly correlated with LC3II expression in patients with sepsis, patients with SAI, and non-surviving patients with SAI. mTOR pathway mediated autophagic-lysosomal disorder showed significant association with CTLA-4 expression.

## Data availability statement

The original contributions presented in the study are included in the article/**Supplementary Material**. Further inquiries can be directed to the corresponding authors.

## Ethics statement

The studies involving humans were approved by the PUMCH institutional review board. The studies were conducted in accordance with the local legislation and institutional requirements. The participants provided their written informed consent to participate in this study.

## Author contributions

WC: Investigation, Methodology, Writing – original draft. JZ: Conceptualization, Methodology, Software, Writing – original draft. DL: Investigation, Resources, Software, Supervision, Visualization, Writing – review & editing. XL: Methodology, Software, Writing – review & editing. HW: Resources, Supervision, Writing – review & editing. NC: Conceptualization, Funding acquisition, Resources, Supervision, Visualization, Writing – review & editing.
