# Supplementary material for: CTLA-4 expression on CD4+ lymphocytes in patients with sepsis-associated immunosuppression and its relationship to mTOR mediated autophagic–lysosomal disorder
Source: Front Immunol. 2024 Jul 22;15:1396157. doi: 10.3389/fimmu.2024.1396157 (PMC11298341; doi:10.3389/fimmu.2024.1396157)
Supplement: Supplementary file 1 [file DataSheet_1.pdf]

CTLA-4 expression on CD4$^+$ lymphocytes in patients with sepsis-associated immunosuppression and its relationship to mTOR mediated autophagic–lysosomal disorder

Additional File S1 Enrollment flowchart

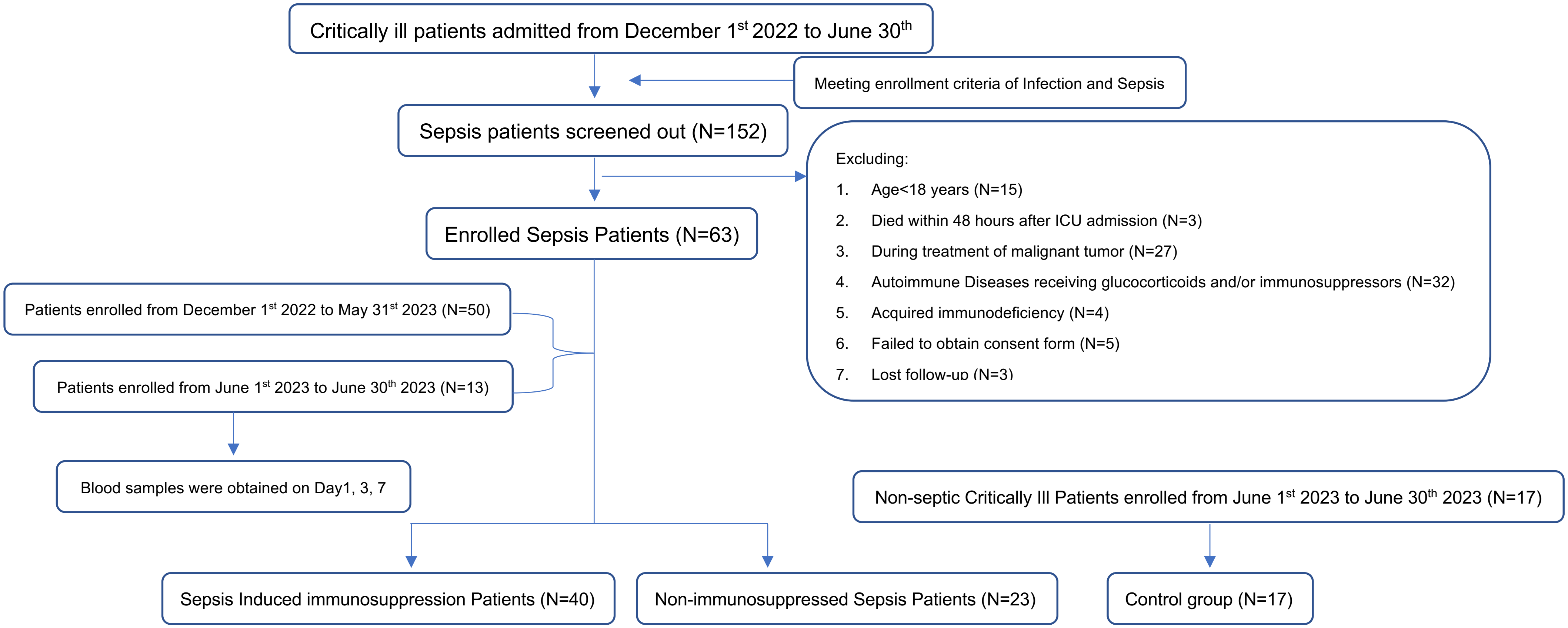

Additional File S2 Comparison between sepsis and non-septic critically ill patients

	All (N=80)	Sepsis (N=63)	Non-sepsis* (N=17)	P
**Baseline characteristics**				
Age	63 (51, 70)	62 (51, 71)	63 (52, 66)	0.632
Sex (Male n %)	46 (57.5%)	36 (57.1%)	10 (58.8%)	0.901
Comorbidities				
Chronic heart disease	37 (46.3%)	22 (34.9%)	15 (88.2%)	<0.0001
COPD	5 (6.3%)	5 (7.9%)	0 (0%)	0.230
Diabetes Mellitus	25 (31.3%)	21 (33.3%)	4 (23.5%)	0.439
Chronic kidney disease	7 (8.8%)	7 (11.1%)	0 (0.0%)	0.150
Chronic liver disease	5 (6.3%)	4 (6.3%)	1 (5.9%)	0.944
Stable Solid Tumor	21 (26.3%)	17 (27.0%)	4 (23.5%)	0.774
Apache II score	17 (12, 25)	19 (16, 28)	8 (6, 12)	<0.0001
SOFA score	7 (5, 11)	8 (5, 11)	5 (4, 6)	0.012
ICU stay	11 (10, 14)	12 (10, 16)	4 (2, 6)	0.002
Hospital Stay	20 (16, 25)	20 (16, 25)	19 (14, 28)	0.274
28-day mortality	18 (22.5%)	18 (28.6%)	0 (0.0%)	0.012
**Vital signs and biochemical tests at admission**				
Temperature (℃)	37.0 (36.3, 37.5)	36.9 (36.2, 37.2)	37.8 (36.9, 38.3)	0.007
Heart Rate (per minute)	88 (77, 98)	87 (75, 98)	91 (89, 96)	0.309
Respiratory Rate (per minute)	16 (15, 20)	16 (14, 20)	16 (15, 20)	0.861
FiO_2 (%)	30 (29, 33)	30 (29, 33)	30 (30, 38)	0.858
PaO_2 (mmHg)	108.0 (89.7, 126.0)	106.5 (88.7, 124.5)	110.0 (93.8, 131.5)	0.544
$PaCO_2$ (mmHg)	39.1 (35.2, 41.8)	39.1 (35.2, 42.1)	39.2 (35.5, 39.8)	0.953
Lactate (mmol/L)	3.6 (2.4, 5.4)	4.2 (2.7, 6.0)	2.0 (1.5, 2.7)	<0.0001
Platelet (*10^9/L)	165 (106, 212)	168 (96, 232)	156 (131, 175)	0.625
Creatinine (umol/L)	84.0 (56.0, 120.0)	83.5 (49.5, 148.5)	86.0 (68.5, 89.5)	0.953
Total Bilirubin (umol/L)	17.1 (12.6, 28.8)	16.9 (11.4, 29.1)	20.9 (14.1, 27.6)	0.248
Albumin (g/L)	33 (31, 36)	32 (30, 35)	36 (34, 38)	0.009
Prothrombin time (s)	13.6 (12.6, 15.1)	13.8 (12.5, 15.3)	13.5 (12.8, 14.2)	0.358
APTT-R	1.12 (0.97, 1.34)	1.18 (1.03, 1.38)	0.97 (0.91, 1.12)	0.012
**T lymphocyte subsets (/ul)**				
White blood cell	11880 (7888, 15520)	11880 (7785, 16930)	11700 (8013, 14375)	0.873
Lymphocyte	639 (431, 1157)	632 (394, 1158)	738 (530, 1161)	0.585
B lymphocyte	120 (63, 207)	119 (63, 216)	120 (76, 192)	0.573
NK T cells	65 (37, 107)	57 (32, 92)	93 (70, 151)	0.041
T lymphocyte	443 (279, 853)	433 (261, 884)	526 (334, 790)	0.969
$CD4^+$ T cell	264 (154, 548)	261 (152, 595)	287 (188, 463)	0.795

$CD8^+$ T cell	158 (106, 244)	145 (92, 246)	167 (134, 235)	0.274
Mem $CD4^+$ T cell	174 (105, 338)	163 (97, 371)	228 (122, 336)	0.585
RAT4	87 (50, 168)	89 (55, 184)	65 (48, 132)	0.585
NaT4	85 (49, 157)	87 (51, 168)	65 (46, 129)	0.585
$CD4^+CD28^+$ T cell	257 (146, 536)	249 (139, 555)	279 (179, 442)	0.730
$CD8^+CD28^+$ T cell	76 (36, 151)	75 (33, 158)	87 (64, 130)	0.969
DRT8	58 (34, 103)	54 (33, 94)	87 (40, 111)	0.101
$CD8^+CD38^+$ T cell	72 (49, 127)	64 (45, 130)	77 (61, 120)	0.585
$CD4^+/CD8^+$ T cell ratio	1.8 (1.3, 2.9)	1.8 (1.2, 2.9)	1.8 (1.2, 2.4)	0.667
**Immunofluorescent staining results on $CD4^+$ T cells**				
IL-2 (%)	11.4 (8.8, 15.9)	11.7 (8.8, 16.3)	10.3 (9.2, 13.7)	0.434
IL-6 (%)	7.5 (5.0, 10.8)	8.2 (5.5, 11.2)	5.1 (4.5, 7.7)	0.029
IL-17 (%)	7.9 (6.2, 11.3)	8.6 (6.4, 12.0)	7.0 (5.4, 8.7)	0.049
TNF-α (%)	10.1 (7.7, 12.3)	10.5 (7.3, 12.6)	9.70 (7.8, 11.7)	0.328
IFN-r (%)	7.4 (4.6, 9.0)	7.4 (4.7, 9.1)	7.2 (3.4, 8.9)	0.516
CTLA-4 (%)	55.1 (42.2, 78.2)	56.7 (41.9, 77.6)	54.5 (40.7, 83.4)	0.905
LC3II (%)	40.6 (21.8, 81.5)	46.5 (25.3, 85.2)	23.7 (6.3, 40.6)	0.029
mTOR (%)	81.3 (60.5, 91.5)	81.5 (60.5, 91.3)	75.8 (59.6, 93.9)	0.585
P62 (%)	95.7 (88.9, 97.6)	95.7 (90.3, 97.6)	94.5 (83.4, 97.6)	0.908
CTLA-4 (MFI)	135.7 (91.5, 335.8)	185.5 (93.8, 366.4)	104.4 (89.6, 128.3)	<0.0001
LC3II-MFI	127.6 (95.9, 196.4)	152.3 (100.2, 236.7)	99.6 (64.5, 118.1)	0.001
mTOR-MFI	150.2 (116.1, 267.5)	166.8 (114.5, 303.3)	143.6 (127.5, 153.8)	0.029
P62-MFI	202.8 (174.0, 316.6)	244.0 (184.3, 340.0)	177.2 (139.6, 190.7)	0.001

* Non-septic patients were those critically ill ones who received major surgery and admitted to our ICU from June 1st 2023 to June 30th 2023.

Values are presented as median and interquartile range (IQR) for continuous variables or as number of cases and percentage for categorical data.

COPD, chronic obstructive pulmonary disease; Apache II score, acute physiology and chronic health evaluation II score; SOFA score, subsequent organ failure assessment score; ICU, intensive care unit; FiO_2, fraction of inspiratory oxygen, PaO_2, partial fraction of arterial oxygen; $PaCO_2$, partial fraction of arterial carbon deoxidate; APTT-R, activated partial thromboplastin time ratio; IL, interleukin; TNF-α, tumor necrosis factor α; IFN-r, interferon r; CTLA-4, cytotoxic T lymphocyte antigen-4; LC3II, microtubule-associated protein light chain 3 type II; mTOR, the mammalian target of rapamycin; MFI, mean fluorescent intensity.

Additional File S3 Comparison of Clinical Parameters and Treatment at ICU Admission between patients with sepsis-associated immunosuppression and non-immunosuppressed sepsis patients

	All sepsis patients N=63	Patients with SAI* N=40	Non-immunosuppressed patients N=23	P
**Clinical Parameters and Treatment at ICU Admission**				
Temperature (℃)	36.9 (36.2, 37.2)	36.9 (36.2, 37.1)	36.8 (36.3, 37.4)	0.791
Heart Rate (per minute)	87 (75, 98)	86 (72, 98)	90 (77, 100)	0.426
Respiratory Rate (per minute)	16 (14, 20)	16 (14, 21)	17 (15, 18)	0.939
FiO_2 (%)	30 (29, 33)	30 (30, 35)	30 (29, 30)	0.606
PaO_2 (mmHg)	106.5 (88.7, 124.5)	107.0 (84.2, 141.0)	104.5 (90.7, 124.0)	0.494
$PaCO_2$ (mmHg)	39.1 (35.2, 42.1)	38.6 (33.5, 42.3)	40.5 (36.5, 42.1)	0.299
Lactate (mmol/L)	4.2 (2.7, 6.0)	4.5 (3.5, 6.3)	3.0 (2.4, 4.5)	0.046
Platelet (*10^9/L)	168 (96, 232)	160 (89, 204)	190 (119, 283)	0.072
Creatinine (umol/L)	83.5 (49.5, 148.5)	103.5 (54.8, 206.5)	73 (46, 96.3)	0.013
Total Bilirubin (umol/L)	16.9 (11.4, 29.1)	17.0 (11.7, 28.7)	15.5 (8.9, 30.1)	0.426
Albumin (g/L)	32 (30, 35)	32 (29, 36)	32 (31, 35)	0.966
Prothrombin Time (S)	13.8 (12.5, 15.3)	13.9 (12.5, 16.2)	13.1 (12.5, 14.3)	0.184
APTT-R	1.2 (1.0, 1.4)	1.2 (1.0, 1.4)	1.1 (1.0, 1.3)	0.791
Procalcitonin (ng/ml)	4.0 (0.9, 19.0)	4.7 (0.7, 22.8)	3.0 (1.0, 8.40)	0.669
(1-3)-β-D-glucan (pg/ml)	24.8 (14.2, 68.8)	32.2 (17.4, 84.4)	12.2 (10.0, 43.6)	0.341
Galactomannan (pg/ml)	0.1 (0.1, 0.2)	0.1 (0.1, 0.2)	0.1 (0.1, 0.2)	0.341
hsCRP (mg/L)	158.1 (81.4, 248.4)	202.8 (83.4, 287)	130.4 (77.8, 199.6)	0.071
**Treatment**				
Mechanical ventilation	56 (88.9%)	36 (90.0%)	20 (87.0%)	0.711
Vasopressors	51 (81.0%)	32 (80.0%)	19 (82.6%)	0.800
Hemodynamic Monitor	7 (11.1%)	6 (15.0%)	1 (4.3%)	0.195
CRRT	18 (28.6%)	15 (37.5%)	3 (13.0%)	0.039
ECMO	2 (3.2%)	1 (2.5%)	1 (4.3%)	0.687
**Initial antibiotics**				
Anti-Gram Positive	26 (41.3%)	20 (50.0%)	6 (26.1%)	0.063
Anti-Gram Negative	63 (100%)	40 (100%)	23 (100%)	1.000
Anti-fungal drugs	21 (33.3%)	19 (47.5%)	2 (8.7%)	0.002
Anti-Virus drugs	5 (7.9%)	3 (7.5%)	2 (8.7%)	0.866

Values are presented as median and interquartile range (IQR) for continuous variables or as number of cases and percentage for categorical data. *SAI, Sepsis-associated immunosuppression, defined as sepsis patients with peripheral lymphocyte counts less than 1000/uL.

ICU, intensive care unit; FiO2, fraction of inspiratory oxygen, PaO2, partial fraction of arterial oxygen; PaCO2, partial fraction of arterial

carbon deoxidate; APTT-R, activated partial thromboplastin time ratio; hsCRP, hypersensitive C-reactive protein; CRRT, continuous renal replacement therapy; ECMO, extracorporeal membrane oxygenation.

Additional File S4 Comparison between survivors and non-survivors in patients with sepsis-associated immunosuppression according to 28-day mortality

	All（N=40）	Non-survivors （N=15）	Survivors （N=25）	P
**Baseline characteristics**				
Age	67 (58, 74)	73 (70, 77)	59 (50, 67)	<0.0001
Sex (Male n %)	21 (52.5%)	9 (60%)	12 (48%)	0.462
Transferred from				0.902
Ward	5 (12.5%)	2 (13.3%)	3 (12%)	
Emergency room	35 (87.5%)	13 (86.7%)	22 (88%)	
Comorbidities				
Chronic heart disease	15 (37.5%)	5 (33.3%)	10 (40%)	0.673
COPD	5 (12.5%)	3 (20%)	2 (8%)	0.267
Diabetes mellitus	13 (32.5%)	5 (33.3%)	8 (32%)	0.931
Chronic kidney disease	7 (17.5%)	3 (20%)	4 (16%)	0.747
Chronic liver disease	3 (7.5%)	0	3 (12%)	0.163
Stable Solid Tumor	12 (30.0%)	7 (46.7%)	5 (20%)	0.075
Infection sites				0.718
Pulmonary	15 (37.5%)	4 (26.7%)	11 (44%)	
Abdominal	20 (50.0%)	9 (60%)	11 (44%)	
Urinary Tract	2 (5.0%)	1 (6.7%)	1 (4%)	
Blood Stream	1 (2.5%0	0	1 (4%)	
Others	2 (5.0%)	1 (6.7%)	1 (4%)	
Apache II score	19 (16, 30)	30 (27, 35)	16 (15, 19)	<0.0001
SOFA score	8 (5, 11)	11 (10, 12)	6 (4, 8)	0.001
ICU stay	13 (11, 18)	12 (11, 13)	13 (11, 23)	0.152
Hospital Stay	21 (16, 26)	17 (14, 23)	22 (19, 27)	0.050
**Clinical Parameters**				
Temperature (C°)	36.9 (36.2, 37.1)	37 (36.3, 37.1)	36.8 (36.2, 37.1)	0.495
Heart Rate (per minute)	86 (72, 98)	82 (71, 96)	87 (77, 99)	0.412
Respiratory Rate (per minute)	16 (14, 21)	15 (14, 21)	17 (14, 21)	0.288
FiO_2 (%)	30 (30, 35)	30 (30, 33)	30 (29.5, 35)	0.934
PaO_2 (mmHg)	107 (84.2, 141)	105 (82, 124)	109 (95.2, 150.5)	0.514
$PaCO_2$ (mmHg)	38.6 (33.5, 42.3)	40 (36.1, 43.3)	37 (32.9, 41.4)	0.514
Lactate (mmol/L)	4.5 (3.5, 6.3)	6.6 (4.5, 9.9)	3.9 (3.3, 5.3)	<0.0001
Platelet (*10^9/L)	160 (89, 204)	155 (74, 204)	165 (92, 210)	0.748
Creatinine (umol/L)	103.5 (54.8, 206.5)	114 (63, 207)	80 (41, 209)	0.514

Total Bilirubin (umol/L)	17 (11.7, 28.7)	21.4 (14.5, 41.1)	16.7 (11.3, 28.5)	0.509
Albumin (g/L)	31.5 (29, 35.8)	34 (29, 36)	31 (29, 33.5)	0.514
Prothrombin Time (s)	13.9 (12.5, 16.2)	15.2 (13.5, 16.4)	13.8 (12.3, 15.7)	0.514
APTT-R	1.2 (1.0, 1.4)	1.3 (1.0, 1.4)	1.2 (1.1, 1.5)	0.514
Procalcitonin (ng/ml)	4.7 (0.7, 22.8)	18 (4.6, 30)	1.8 (0.6, 19.5)	0.050
(1-3)-β-D-glucan (pg/ml)	32.2 (17.4, 84.4)	53.4 (16.8, 207.4)	29.5 (18.6, 66.5)	0.155
Galactomannan (pg/ml)	0.1 (0.1, 0.2)	0.1 (0.1, 0.2)	0.1 (0.1, 0.2)	0.542
hsCRP (mg/L)	202.8 (83.4, 287)	240.1 (109.8, 314.8)	156.3 (67.2, 280.4)	0.094
Treatment				
Mechanical ventilation	36 (90.0%)	13 (86.7%)	23 (92%)	0.586
Vasopressors	32 (80.0%)	13 (86.7%)	19 (76%)	0.414
Hemodynamic Monitor	6 (15.0%)	2 (13.3%)	4 (16%)	0.819
CRRT	15 (37.5%)	7 (46.7%)	8 (32%)	0.354
ECMO	1 (2.5%)	0	1 (4.0%)	0.433
Initial Antibiotics				
Anti-Gram-Positive drugs	20 (50.0%)	8 (53.3%)	12 (48%)	0.744
Anti-Gram-Negative drugs	40 (100%)	15 (100%)	25 (100%)	1.000
Anti-fungal drugs	19 (47.5%)	9 (60%)	10 (40%)	0.220
Anti-Virus drugs	3 (7.5%)	1 (6.7%)	2 (8.0%)	0.877
**T lymphocyte subsets (/uL) (median and IQR)**				
White blood cell	9260 (6620, 14900)	11650 (7190, 18860)	8305 (4645, 12605)	0.149
Lymphocyte	445 (360, 613)	400 (370, 477)	477 (348, 647)	0.191
B lymphocyte	74 (44, 118)	64 (40, 113)	79 (55, 123)	0.514
NK T cells	42 (19, 60)	44 (19, 65)	40 (15, 60)	0.920
T lymphocyte	305 (224, 416)	283 (219, 372)	326 (235, 475)	0.514
$CD4^+$ T cell	178 (134, 257)	157 (137, 241)	194 (116, 305)	0.514
$CD8^+$ T cell	108 (74, 145)	108 (79, 117)	107 (72, 169)	0.363
$CD4^+CD28^+$ T cell	166 (104, 233)	146 (108, 194)	180 (100, 288)	0.514
$CD8^+CD28^+$ T cell	38 (23, 76)	33 (24, 39)	64 (20, 91)	0.050
$CD8^+CD38^+$ T cell	56 (33, 84)	56 (33, 82)	56 (33, 87)	0.934
$CD4^+/CD8^+$ T ratio	1.7 (1.1, 2.9)	1.5 (1.1, 2.7)	1.8 (1.1, 3.1)	0.514
**Markers on $CD4^+$ T cells (median and IQR)**				
IL-2 (%[¥])	9.9 (8.1, 15.6)	12.4 (7.6, 16.7)	9.6 (8.2, 14.9)	0.283
IL-6 (%)	7.9 (5.8, 10.8)	6.8 (5.4, 10.0)	9.0 (5.8, 11.1)	0.514
IL-17 (%)	7.9 (5.9, 10.4)	9.3 (8.4, 12.3)	6.5 (5.6, 9.5)	0.009
TNF-α (%)	9.4 (6.9, 12.2)	7.1 (6.7, 11.1)	10.5 (8.2, 13.4)	0.044
IFN-r (%)	6.8 (4.4, 8.7)	7.9 (4.3, 9.4)	6.8 (4.6, 8.0)	0.404
CTLA-4 (MFI)	328.7 (188.6, 440.8)	506.7 (366.4, 571.5)	225.2 (152.0, 307.9)	<0.0001

CTLA-4 (%)	65.9 (49.7, 85.9)	90.8 (74.4, 93.4)	56.7 (39.8, 64.4)	<0.0001
LC3I (%)	28.4 (10.4, 66.1)	19.2 (5.71, 32.6)	37.5 (12.6, 73.5)	0.050
LC3I-MFI	211.0 (152.5, 303.4)	170.7 (125.2, 225.5)	277.7 (158.3, 320.1)	0.050
LC3II (%)	55.9 (29.6, 88.8)	88.8 (59.8, 91.9)	37.7 (18.2, 70.9)	0.009
LC3II-MFI	184.7 (105.7, 266.3)	265.1 (199.0, 312.5)	145.9 (78.3, 220.2)	0.01
mTOR (%)	81.9 (65.9, 92.7)	81.3 (77.1, 88.9)	82.4 (40.2, 93.2)	0.092
mTOR-MFI	267.2 (139.8, 360.8)	266.7 (185.9, 339.6)	267.8 (117.6, 362.3)	0.623
P62 (%)	96.2 (93.5, 97.8)	96.9 (91.4, 97.9)	96.1 (94.3, 97.7)	0.631
P62-MFI	314.8 (250.8, 367.6)	340.1 (249.2, 391.9)	311.2 (259.9, 363.2)	0.532

Values are presented as median and interquartile range (IQR) for continuous variables or as number of cases and percentage for categorical data. *Sepsis-associated immunosuppression was defined as sepsis patients with peripheral lymphocyte counts less than 1000/uL. ¥ means proportions of positive $CD4^+$ lymphocytes.

COPD, chronic obstructive pulmonary disease; Apache II score, acute physiology and chronic health evaluation II score; SOFA score, subsequent organ failure assessment score; ICU, intensive care unit; FiO2, fraction of inspiratory oxygen, PaO2, partial fraction of arterial oxygen; PaCO2, partial fraction of arterial carbon deoxidate; APTT-R, activated partial thromboplastin time ratio; hsCRP, hypersensitive C-reactive protein; CRRT, continuous renal replacement therapy; ECMO, extracorporeal membrane oxygenation; NK T cell, natural killer T cell; IL, interleukin; TNF-α, tumor necrosis factor α; IFN-γ, interferon γ; CTLA-4, cytotoxic T lymphocyte antigen-4; LC3I, microtubule-associated protein light chain 3 type I; LC3II, microtubule-associated protein light chain 3 type II; mTOR, the mammalian target of rapamycin; MFI, mean fluorescent intensity.

Additional File S5 Comparison of marker MFIs on $CD4^{+}$ lymphocyte between survivors and non-survivors of patients with sepsis-associated immunosuppression.

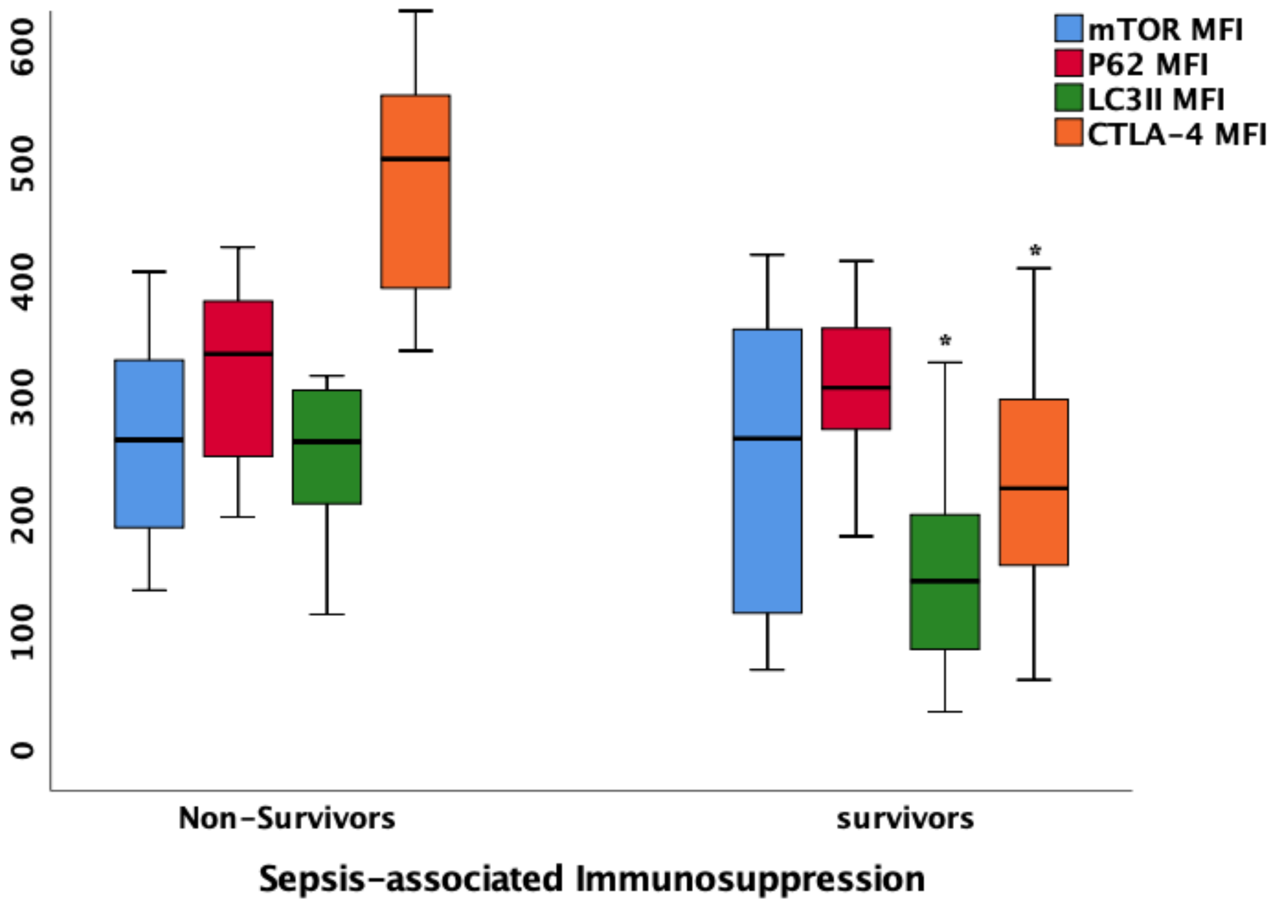

The MFI of LC3II and CTLA-4 were significantly different between survivors and non-survivors of patients with sepsis-associated immunosuppression.

Survivors and non-survivors were stratified according to 28-day mortality. Black Asteroids meant that the comparison between the two groups was significant with $P<0.05$.

MFI, mean fluorescence intensity; mTOR, Mammalian target of rapamycin; LC3II, microtubule-associated protein light chain 3 type II; CTLA-4, cytotoxic T lymphocyte antigen-4.

Additional File S6 Comparison between survivors and non-survivors of sepsis patients according to 28-day mortality

	All (N=63)	Non-survivors (n=18)	Survivors (n=45)	P
**Baseline characteristics**				
Immunosuppressed (%)	40 (63.5%)	15 (83.3%)	25 (55.6%)	0.039
Age	62 (51, 71)	71 (70, 77)	58 (48, 67)	<0.0001
Sex (Male n %)	36 (57.1%)	12 (66.7%)	24 (53.3%)	0.334
Transferred from				0.649
Ward	9 (14.3%)	2 (11.1%)	7 (15.6%)	
Emergency Room	54 (85.7%)	16 (88.9%)	38 (84.4%)	
Comorbidities				
Congestive heart disease	22 (34.9%)	5 (27.8%)	17 (37.8%)	0.452
COPD	5 (7.9%)	3 (16.7%)	2 (4.4%)	0.105
Diabetes mellitus	21 (33.3%)	6 (33.3%)	15 (33.3%)	1.000
Chronic Kidney Disease	7 (11.1%)	3 (16.7%)	4 (8.9%)	0.375
Chronic Liver Disease	4 (6.3%)	0 (0%)	4 (8.9%)	0.191
Stable Solid Tumor	17 (27.0%)	7 (38.9%)	10 (22.2%)	0.178
Infection sites				0.528
Pulmonary	21 (33.3%)	4 (22.2%)	17 (37.8%)	
Abdominal	33 (52.4%)	12 (66.7%)	21 (46.7%)	
Urinary Tract	3 (4.8%)	1 (5.6%)	2 (4.4%)	
Blood Stream	3 (4.8%)	0 (0%)	3 (6.7%)	
Others	3 (4.8%)	1 (5.6%)	2 (4.4%)	
Apache II score	19 (16, 28)	30 (25, 35)	17 (14, 22)	<0.0001
SOFA score	8 (5, 11)	11 (10, 12)	7 (5, 11)	0.002
ICU stay	12 (10, 16)	12 (10, 13)	12 (11, 17)	0.779
Hospital Stay	20 (16, 25)	18 (14, 21)	21 (18, 26)	0.015
**Clinical Parameters**				
Temperature (**C°)**	36.9 (36.2, 37.2)	37 (36.3, 37.1)	36.8 (36.2, 37.2)	0.940
Heart Rate (per minute)	87 (75, 98)	80 (71, 92)	88 (77, 100)	0.088
Respiratory Rate (per minute)	16 (14, 20)	16 (14, 21)	17 (15, 20)	0.326
FiO_2 (%)	30 (29, 33)	30 (29.5, 33)	30 (29, 33)	0.655
PaO_2 (mmHg)	106.5 (88.7, 124.5)	106 (82.1, 124)	107 (92.6, 126.5)	0.743
$PaCO_2$ (mmHg)	39.1 (35.2, 42.1)	40.5 (37.1, 42.9)	38.2 (35.1, 41.7)	0.255
Lactate (mmol/L)	4.2 (2.7, 6.0)	6.9 (4.5, 9.9)	3.6 (2.5, 4.8)	0.001
Platelet ($*10^9$/L)	168 (96, 232)	162 (104, 196)	168 (96, 251)	0.797
Creatinine (umol/L)	83.5 (49.5, 148.5)	106 (63, 187.5)	77 (44.5, 140)	0.569
Total Bilirubin (umol/L)	16.9 (11.4, 29.1)	21.4 (14.7, 38.9)	16.2 (10.9, 28.5)	0.255
Albumin (g/L)	32 (30, 35)	33 (30, 36)	32 (31, 35)	0.529
Prothrombin Time (s)	13.8 (12.5, 15.3)	14.8 (12.9, 16.4)	13.5 (12.5, 14.8)	0.569
APTT-R	1.2 (1.0, 1.4)	1.3 (1.0, 1.4)	1.2 (1.0, 1.4)	0.569
Procalcitonin (ng/ml)	4.0 (0.9, 19.0)	16.5 (4.2, 27)	2.7 (0.7, 13.5)	0.010
(1-3)-β-D-glucan (pg/ml)	24.8 (14.2, 68.8)	23.9 (11.9, 182.7)	24.8 (14.3, 58.9)	0.842
Galactomannan (pg/ml)	0.1 (0.1, 0.2)	0.1 (0.1, 0.2)	0.1 (0.1, 0.2)	0.720

hsCRP (mg/L)	158.1 (81.4, 248.4)	228.5 (170.1, 320.2)	130.4 (75.9, 223.2)	0.002
Treatment				
Mechanical ventilation	56 (88.9%)	16 (88.9%)	40 (88.9%)	1.000
Vasopressors	51 (81.0%)	16 (88.9%)	35 (77.8%)	0.310
Hemodynamic Monitor	7 (11.1%)	2 (11.1%)	5 (11.1%)	1.000
CRRT	18 (28.6%)	8 (44.4%)	10 (22.2%)	0.078
ECMO	2 (3.2%)	0 (0%)	2 (4.4%)	0.363
Initial Antibiotics				
Anti-Gram-Positive drugs	26 (41.3%)	8 (44.4%)	18 (40.0%)	0.746
Anti-Gram-Negative drugs	63 (100%)	18 (100%)	45 (100%)	1.000
Anti-fungal drugs	21 (33.3%)	9 (50.0%)	12 (26.7%)	0.076
Anti-Virus drugs	5 (7.9%)	1 (5.6%)	4 (8.9%)	0.658
**T lymphocyte subsets (/uL) (median and IQR)**				
WBC	11880 (7785, 16930)	11650 (8225, 17730)	11990 (7770, 17040)	0.683
Lymphocyte	632 (394, 1158)	425 (375, 643)	797 (465, 1312)	0.015
B lymphocyte	119 (63, 216)	83 (42, 190)	139 (74, 253)	0.189
NK T cells	57 (32, 92)	49 (28, 74)	60 (32, 100)	0.247
T lymphocyte	433 (261, 884)	315 (227, 423)	597 (303, 900)	0.028
$CD4^+$ T cell	261 (152, 595)	180 (145, 269)	401 (172, 612)	0.031
$CD8^+$ T cell	145 (92, 246)	110 (81, 173)	179 (95, 306)	0.061
$CD4^+CD28^+$ T cell	249 (139, 555)	167 (1221, 249)	357 (164, 569)	0.015
$CD8^+CD28^+$ T cell	75 (33, 158)	36 (26, 60)	94 (42, 169)	0.003
$CD8^+CD38^+$ T cell	64 (45, 130)	58 (35, 94)	74 (47, 142)	0.449
$CD4^+/CD8^+$ T ratio	1.8 (1.2, 2.9)	1.7 (1.3, 2.9)	1.8 (1.2, 2.9)	0.842
**Markers on $CD4^+$ T cells (median and IQR)**				
IFN-γ (%¥)	7.4 (4.7, 9.1)	7.7 (4.1, 9.7)	7.4 (5.6, 8.8)	0.720
IL-2 (%)	11.7 (8.8, 16.3)	11.8 (7.5, 17.1)	11.7 (8.8, 15.6)	0.842
IL-6 (%)	8.2 (5.5, 11.2)	6.5 (5.3, 10.3)	8.7 (5.8, 11.9)	0.189
IL-17 (%)	8.6 (6.4, 12.0)	9.2 (7.2, 12.3)	7.8 (6.1, 11.6)	0.140
TNF-α (%)	10.5 (7.3, 12.6)	7.5 (6.8, 11.2)	10.7 (8.3, 13.9)	0.449
CTLA-4 (MFI)	185.5 (93.8, 366.4)	427.5 (346.9, 555.8)	130.6 (65.8, 230.1)	0.002
CTLA-4 (%)	56.7 (41.9, 77.6)	90.2 (73.2, 93.5)	50.3 (32.7, 59.1)	<0.0001
LC3I (%)	28.1 (12.9, 55.6)	19 (8.15, 33.1)	36.2 (13.7, 69.9)	0.061
LC3I-MFI	146.1 (82.2, 249.1)	168.6 (101.4, 195.5)	125.9 (79.8, 285.9)	0.720
LC3II (%)	46.5 (25.3, 85.2)	87.7 (64.8, 91.3)	37.8 (19.4, 63.0)	<0.0001
LC3II-MFI	152.3 (100.2, 236.7)	244.3 (181.1, 307.3)	122.8 (90.1, 166.9)	<0.0001
mTOR (%)	81.5 (60.5, 91.3)	82.5 (74.9, 91.4)	81.5 (55.5, 91.5)	0.842
mTOR-MFI	166.8 (114.5, 303.3)	227.7 (161.5, 332.4)	140.9 (103.4, 294.4)	0.078
P62 (%)	95.7 (90.3, 97.6)	96.4 (90.9, 98.0)	95.7 (88.8, 97.5)	0.720
P62-MFI	244.0 (184.3, 340.0)	302.9 (225.0, 381.7)	208.9 (174.3, 319.7)	0.039

Values are presented as median and interquartile range (IQR) for continuous variables or as number of cases and percentage for categorical data. *Sepsis-associated immunosuppression was defined as sepsis patients with peripheral lymphocyte counts less than 1000/uL. ¥ means proportions of positive $CD4^+$ lymphocytes.

COPD, chronic obstructive pulmonary disease; Apache II score, acute physiology and chronic health evaluation II score; SOFA score, subsequent organ failure assessment score; ICU, intensive care unit; FiO2, fraction of inspiratory oxygen, PaO2, partial fraction of arterial oxygen; PaCO2, partial fraction of arterial carbon deoxidate; APTT-R, activated partial thromboplastin time ratio; hsCRP, hypersensitive C-reactive protein; CRRT, continuous renal replacement therapy; ECMO, extracorporeal membrane oxygenation; WBC, white blood cell; NK T cell, natural killer T cell; IL, interleukin; TNF-α, tumor necrosis factor α; IFN-γ, interferon γ; CTLA-4, cytotoxic T lymphocyte antigen-4; LC3I, microtubule-associated protein light chain 3 type I; LC3II, microtubule-associated protein light chain 3 type II; mTOR, the mammalian target of rapamycin; MFI, mean fluorescent intensity.

Additional file S7 Receiver operating characteristic (ROC) curve for predicting the 28-day mortality of sepsis patients

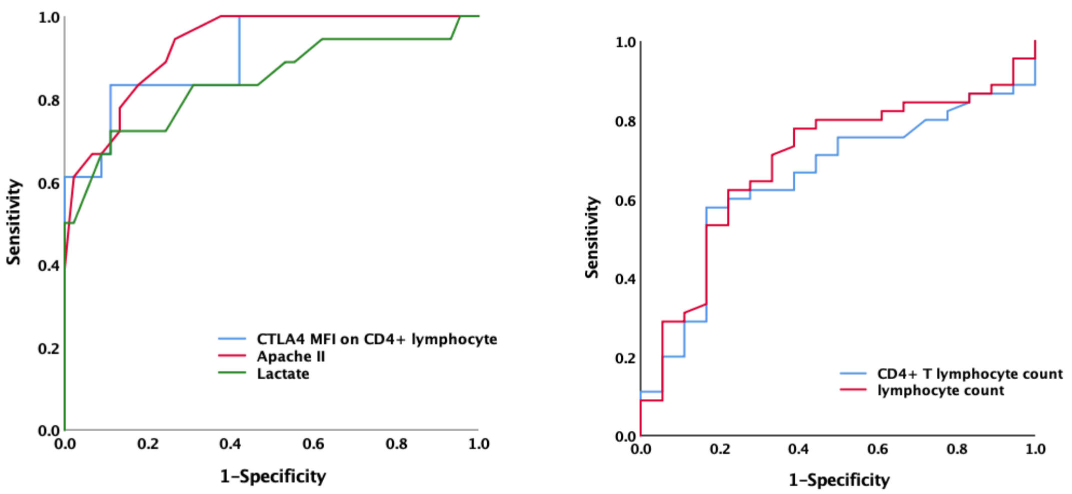

AUCs: MFI of CTLA-4 on CD4+ T cells 0.906, Apache II score 0.929, Lactate level 0.8411, peripheral Lymphocyte count 0.650, CD4+ T cell count 0.693. A cutoff value of CTLA-4 MFI 328.7 at ICU admission was able to predict prognosis with a sensitivity of 83.3% and specificity of 89.9% (AUC 0.906).

AUC, area under the curve; MFI, mean fluorescence intensity; CTLA-4, cytotoxic T lymphocyte antigen-4; Apache II score, acute physiology and chronic health evaluation II score.

Additional file S8 Flow cytograms showing the gating strategy of P62, LC3B, p-mTOR and CTLA-4 and their comparison with unstained samples (Isotype)

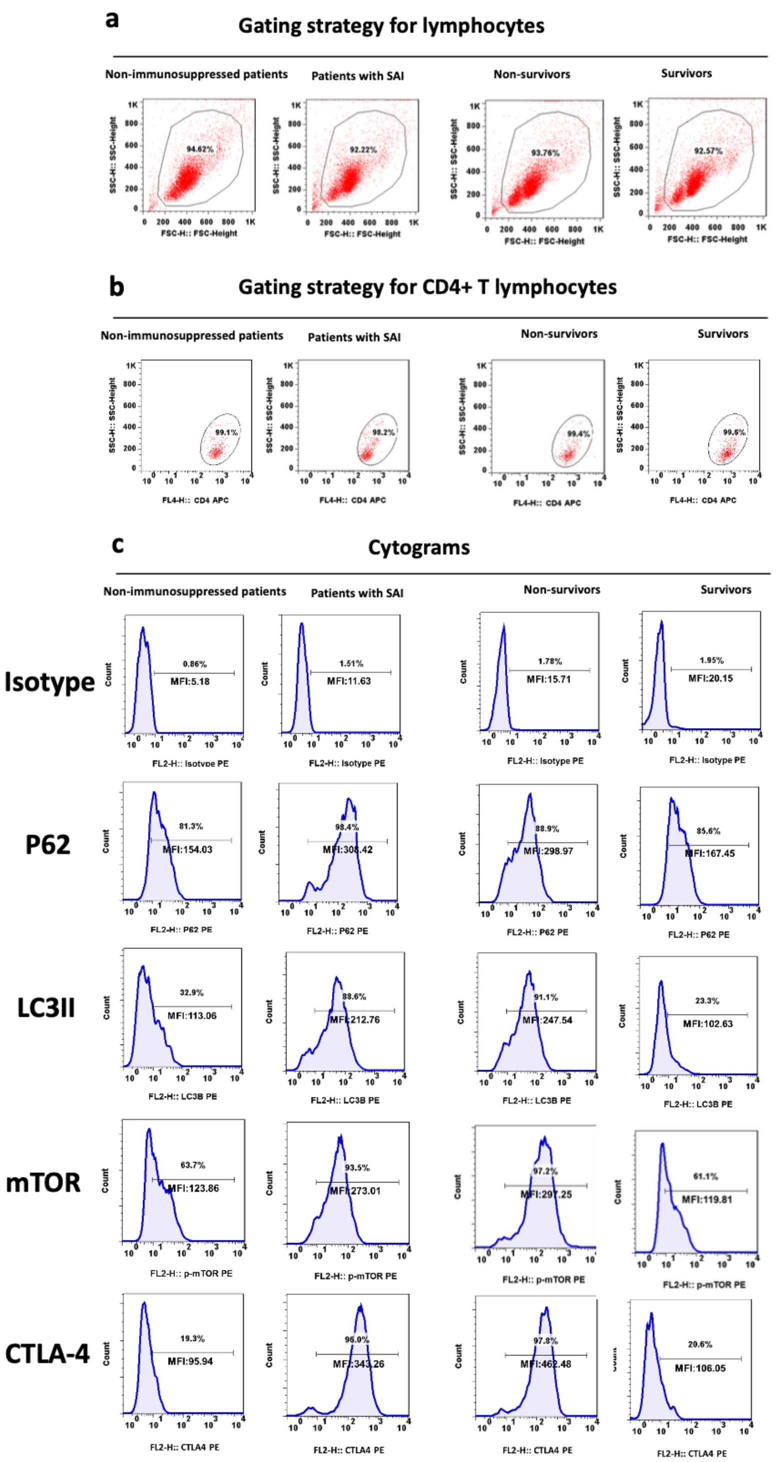

CTLA-4, cytotoxic T lymphocyte antigen-4; LC3I, microtubule-associated protein light chain 3 type I; LC3II, microtubule-associated protein light chain 3 type II; mTOR, the mammalian target of rapamycin; MFI, mean fluorescent intensity.

Additional File S9 Dynamic changes of CTLA-4 MFI on CD4+ lymphocyte, peripheral lymphocyte count and CD4+ T cell count in sepsis patients.

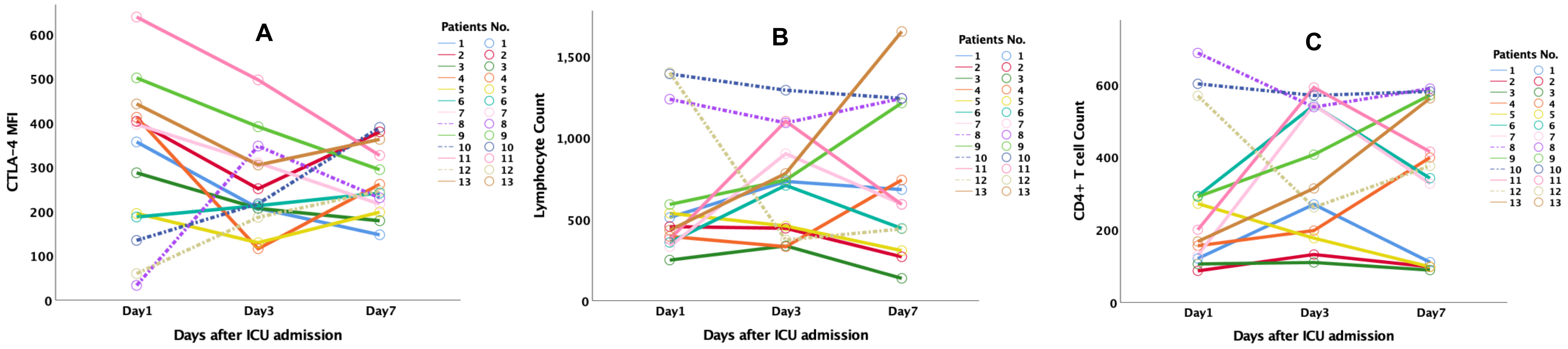

13 patients were monitored dynamically, and 10 patients were diagnosed with sepsis-associated immunosuppression according to our grouping criteria. Dash line: non-immunosuppressed sepsis patients; Solid line: patients with sepsis-associated immunosuppression.

A: Dynamic changes of CTLA-4 MFI on CD4+ lymphocyte in sepsis patients

B: Dynamic changes of peripheral lymphocyte count in sepsis patients

C: Dynamic changes of CD4+ T cell count in sepsis patients

CTLA-4, cytotoxic T lymphocyte antigen-4; MFI, mean fluorescent intensity.
